# Elucidating the constitutive relationship of calcium–silicate–hydrate gel using high throughput reactive molecular simulations and machine learning

**DOI:** 10.1038/s41598-020-78368-1

**Published:** 2020-12-07

**Authors:** Gideon A. Lyngdoh, Hewenxuan Li, Mohd Zaki, N. M. Anoop Krishnan, Sumanta Das

**Affiliations:** 1 Department of Civil and Environmental Engineering, University of Rhode Island, Kingston, RI USA; 2 Department of Mechanical, Industrial and Systems Engineering, University of Rhode Island, Kingston, RI USA; 3 Department of Civil Engineering, Indian Institute of Technology Delhi, Hauz Khas, New Delhi, 110016 India; 4 Department of Materials Science and Engineering, Indian Institute of Technology Delhi, Hauz Khas, New Delhi, 110016 India

**Keywords:** Structural materials, Theory and computation

## Abstract

Prediction of material behavior using machine learning (ML) requires consistent, accurate, and, representative large data for training. However, such consistent and reliable experimental datasets are not always available for materials. To address this challenge, we synergistically integrate ML with high-throughput reactive molecular dynamics (MD) simulations to elucidate the constitutive relationship of calcium–silicate–hydrate (C–S–H) gel—the primary binding phase in concrete formed via the hydration of ordinary portland cement. Specifically, a highly consistent dataset on the nine elastic constants of more than 300 compositions of C–S–H gel is developed using high-throughput reactive simulations. From a comparative analysis of various ML algorithms including neural networks (NN) and Gaussian process (GP), we observe that NN provides excellent predictions. To interpret the predicted results from NN, we employ SHapley Additive exPlanations (SHAP), which reveals that the influence of silicate network on all the elastic constants of C–S–H is significantly higher than that of water and CaO content. Additionally, the water content is found to have a more prominent influence on the shear components than the normal components along the direction of the interlayer spaces within C–S–H. This result suggests that the in-plane elastic response is controlled by water molecules whereas the transverse response is mainly governed by the silicate network. Overall, by seamlessly integrating MD simulations with ML, this paper can be used as a starting point toward accelerated optimization of C–S–H nanostructures to design efficient cementitious binders with targeted properties.

## Introduction

In recent years, the quest for new and emerging high-performance materials has been increasing rapidly in the fields of infrastructure, aviation, energy, and communications. To address this challenge, machine learning (ML)-based approaches have emerged as promising avenues to accelerate the development of innovative materials design strategies^1–3^. Fundamental evaluation of composition-property relationships in highly heterogeneous systems is a key feature of such materials design strategies. ML, when judiciously used, can learn various complex composition-property relationships that would otherwise remain undetected using traditional approaches^4,5^. However, the application of such ML-based approaches is still limited, especially in the field of infrastructure materials^6,7^. It is critical to find bold and forward-thinking solutions in infrastructure that adopt modern methodologies for materials design and discovery so as to accelerate the development of next-generation of durable, high-performance materials.

Ordinary Portland cement (OPC) concrete is the most widely used construction material. Despite vast research on composition-property relationships over the last 3 decades^8–11^, the influence of the heterogeneous hierarchical structure of the material on the engineering performance still remains an active area of research^8,9^. Specifically, previous studies have highlighted that the mechanical performance and, durability of cementitious materials can be improved by optimizing the properties of calcium–silicate–hydrate (C–S–H) gel—the glue of concrete formed via hydration of cement^11,12^. C–S–H exhibits a poorly crystalline structure as observed from scattering experiments^13,14^. While fundamental composition–property relationships for C–S–H are crucial towards the design of high-performance and high-durability concrete via “bottom-up” approach^15^, complex hierarchical characteristics of C–S–H makes it exceedingly difficult to probe such relationships of C–S–H experimentally^14,16^.

To this extent, ML approaches are a promising solution to predict composition–property relationships toward the design of cementitious materials. However, evaluation and prediction of such relationships for C–S–H gel using ML present various well-known challenges. First, ML algorithms critically rely on the existence of accessible, consistent, accurate, and, representative datasets to provide enough information for training the models. Such large experimental data for C–S–H are limited or clustered to a few feasible regions. Second, ML, being a data-driven method, doesn’t provide insights into the fundamental laws of physics and, therefore, can potentially result in non-physical solutions^4,5^. Specifically, the black box ML methods such as NN, despite having high predictability, have little or no interpretability. To overcome these challenges, in this paper, we adopt a systematic and pragmatic approach where high-throughput molecular dynamics (MD) simulation is synergistically integrated with various advanced ML techniques especially Gaussian process (GP) and neural network (NN) to evaluate composition-dependent elastic moduli of C–S–H. Besides, various other ML techniques such as polynomial regression (PR), random forest (RF), support vector machine (SVM), k-nearest neighbors (k-NN), and decision trees (DT) are also evaluated for a comparative overview. Further, the interpretability of the black box models are explored using shapley additive explanations (SHAP)^17,18^ to gain insights into the fundamental factors governing the elastic response of C–S–H.

Precisely, a composition-dependent elastic constant database for C–S–H is developed using high-throughput MD simulation. MD simulations have been exhaustively used to investigate the structure of C–S–H^19–22^, exploring information that are not feasible in traditional experiments, despite recent advances in characterization. Such, MD-simulation-based database generation follows fundamental laws of physics and thus, helps to avoid non-physical solutions. However, the accuracy of MD simulations depends on the choice of interatomic potential. Here, reactive forcefield (ReaxFF)^23^ has been adopted which has been shown to yield a good correlation between the simulated and experimental responses of C–S–H^21,24^. While a large dataset is generated using physics-based MD simulations, supervised ML techniques are leveraged which explore the information by learning a pattern from the data generated by MD simulations. As discussed earlier, the application of ML techniques on cementitious materials is limited. A few studies^6,7,25^ have applied various ML techniques on experimental compressive strength data for concrete at the macro-scale. While these studies addressed the macro-scale relationship of a single target (such as compressive strength) from multiple inputs such as change of mixture proportions or starting materials for concrete, this paper evaluates multiple elastic constants (C_11_, C_22_, C_33_, C_44_, C_55_, C_66_, C_12_, C_13_, and C_23_ ) of the primary binding phase (C–S–H) with varying fraction of CaO, SiO_2,_ and nanoconfined water.

To tackle such a multi-target problem, this paper employs both multiple single target (ST) approach (for PR, RF, SVM, k-NN, DT, and GP) and multi-target regression approach (for NN). While multiple single target (ST) regression splits the problem into multiple single-output regression problems where the outputs are assumed to be independent of each other, multi-target regression incorporates the statistical correlation among the outputs besides using the original input features. As such, multi-target regression is likely to offer superior response predictions for C–S–H due to its multivariate nature and the compound dependencies between the multiple feature and/or target variables^26,27^ which is explored in detail in this paper. Though NN can provide high accuracy of prediction, interpretation of results with NN alone challenging and it may not offer any new physical insights^4,28^. Along those lines, this study adopts a recent method called SHAP^17,18^ to address this challenging issue of interpretation of results from NN model. Overall, this paper, aimed at predicting composition-dependent multiple elastic constants for C–S–H, is expected to provide a valuable composition-property link for C–S–H which can help clarify efficient pathways to optimize the nanoscale C–S–H structures to enhance mechanical performance and, durability of cementitious materials.

## Results

### MD simulations to generate large dataset

**Figure 1:**
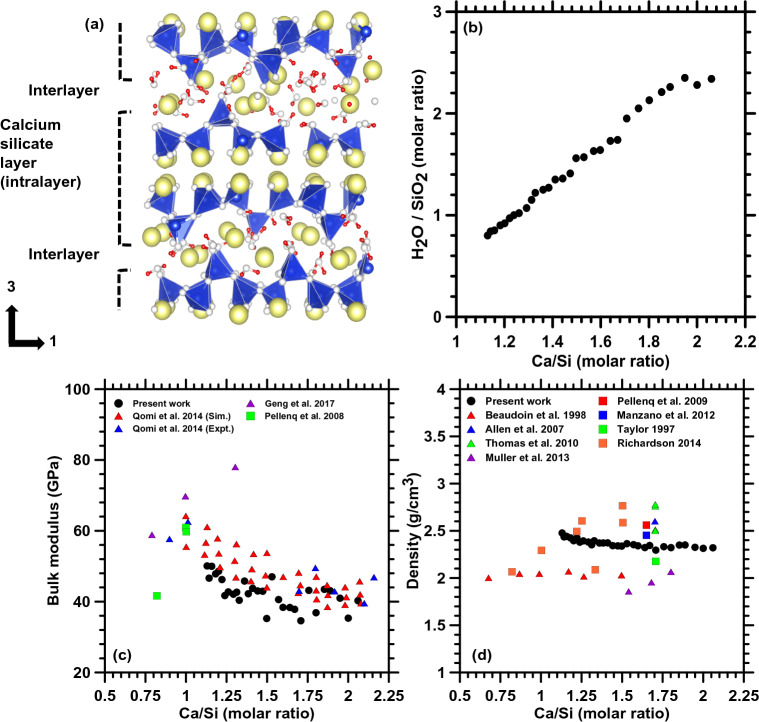
(**a**) Representative C–S–H structure for Ca/Si = 1.09 showing the Calcium silicate network and the interlayer spaces, and (**b**) water content (H_2_O/SiO_2_ molar ratio) as a function of Ca/Si molar ratio for representative C–S–H structures with saturation water content, (**c**) The bulk modulus, and (**d**) density as a function of Ca/Si molar ratio for representative C–S–H structures with saturation water content.

A total of 319 different C–S–H compositions are generated via MD simulations by varying the CaO, SiO_2,_ and water content. C–S–H has been reported extensively in the literature^16,19,20^ to exhibit a layered structure. It consists of interlayer domains in between calcium silicate networks that contain water molecules. While Fig. 1a shows a representative atomistic structure of C–S–H with a Ca/Si ratio of 1.09, Fig. 1b plots the variations of water content as a function of Ca/Si molar ratio. The model construction process and relevant details are provided in the methods section. Figure 1b clearly shows a significant increase in water content with an increasing Ca/Si ratio. Such trend can be attributed to the increase in the degree of depolymerization and increase in interlayer spacing with increasing CaO content in C–S–H. A similar observation has also been reported in the literature^20^.

Figure 1c and d shows the computed bulk modulus and density respectively for C–S–H plotted with varying Ca/Si ratio. The values obtained from MD simulations in this present study are compared with experimental results available in the literature^11,22,23,29–35^. It is observed that the computed bulk modulus values are in good agreement with the experimental values which provides confidence in the reliability of the constructed C–S–H structures. In Fig. 1d, it is observed that the experimental density values, obtained from literature, are scattered within a large range which can be attributed to the process by which the hydrated samples were dried under various environmental conditions^36^. The densities obtained from MD simulations in the present work lie within the experimentally observed range. The general trend in Fig. 1c and d suggest that both bulk modulus and density decrease with an increase in the Ca/Si ratio. The influence of the composition of C–S–H on the elastic constants, as obtained from MD simulations, is detailed hereafter in the remainder of this section.

**Figure 2:**
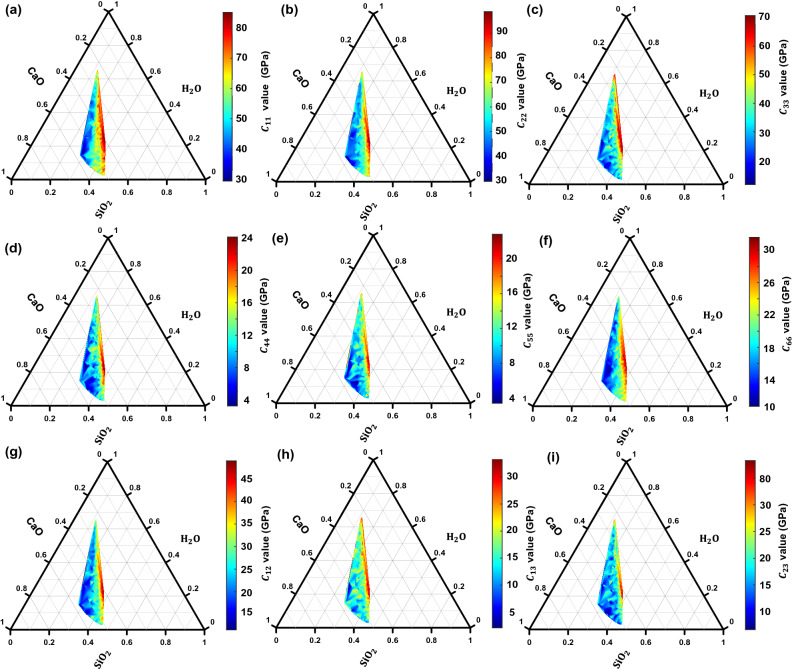
Ternary diagram showing the stiffness moduli (**a**) C_11_, (**b**) C_22_, (**c**) C_33_, (**d**) C_44_, (**e**) C_55_, (**f**) C_66_, (**g**) C_12_, (**h**) C_13_, and (**i**) C_23_ values obtained via MD simulations with varying CaO–SiO_2_–H_2_O molar fractions.

**Figure 3:**
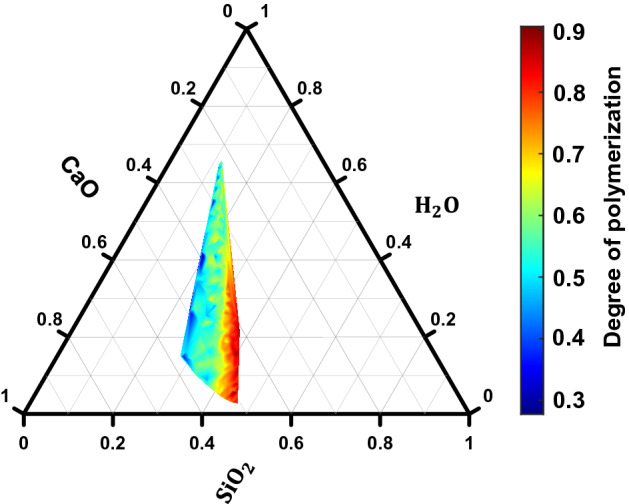
Ternary diagram showing the influence of CaO–SiO_2_–H_2_O content on the degree of polymerization.

Figure 2 shows the ternary plot of elastic constants with respect to the CaO, SiO_2_, and H_2_O present in different C–S–H compositions. The general trend from the figures suggests that for the same concentration of water, the modulus decreases with an increase in CaO concentration. This is due to the fact that as the content of CaO concentration increases, the structure becomes more disordered, and depolymerization of the network structure increases (as shown in Fig. 3) resulting in a decrease in the elastic modulus. On the other hand, an increase in H_2_O concentration for constant molar fraction of CaO results in a decrease in the modulus value. However, with an increase in H_2_O concentration and the same molar fraction of SiO_2_ the elastic modulus increase. It can be observed that the variation of moduli with composition is non-systematic and coupled effects exist. For example, C_33_ value initially increases with an increase in SiO_2_ content up to a 0.4 molar fraction beyond which the value decreases with a further increase in water concentration. Similarly, from Fig. 2, it is evident that C_11_ and C_22_ are greater than C_33_. This is due to the presence of interlayer spacing in layered C–S–H structure where the load is applied perpendicular to the interlayer plane. Similarly, for the same reason, in the case of shear deformation C_66_ values are found to be higher than C_44_ and C_55_. Overall, the stiffness moduli exhibit a non-linear relationship with variations in composition, which prevents any assumption of a linear model to predict the stiffness moduli in the C–S–H system.

In general, the elastic modulus (or Young’s modulus) increases with increasing network connectivity^37^. To evaluate such a trend in this study, the connectivity in the structure is calculated by computing the degree of polymerization which is taken as the ratio of the number of BO (bridging oxygen) with respect to the number of tetrahedral networks (T). The ternary plot of the degree of polymerization with the composition of C–S–H is shown in Fig. 3. A higher degree of polymerization is observed when the concentration of SiO_2_ increases which is expected since Si serves as a network former in C–S–H. Conversely, a lower degree of polymerization is observed when water concentration is increased and SiO_2_ molar fraction is decreased. However, the trend reverses when the water content increases and CaO content decreases. This infers the existence of coupled effect in composition-structure properties in C–S–H, which is also observed for elastic moduli. Maximum network connectivity is observed when the SiO_2_ molar fraction is greater than 0.4 and the H_2_O molar fraction is below 0.1. But maximum elastic moduli are observed in the range between 0.2 and 0.4 molar fraction for both SiO_2_ and H_2_O. This indicates that the network connectivity alone is not sufficient enough to predict the elastic constants which makes it challenging to develop a robust physics-based predictive model.

### Prediction of elastic constants using ML

While the MD simulations are leveraged to obtain a database of elastic constants for C–S–H as explained in the previous section, the forthcoming sub-sections use that database and implement various ML approaches to build prediction tools for elastic constants for C–S–H as explained hereafter.

**Figure 4:**
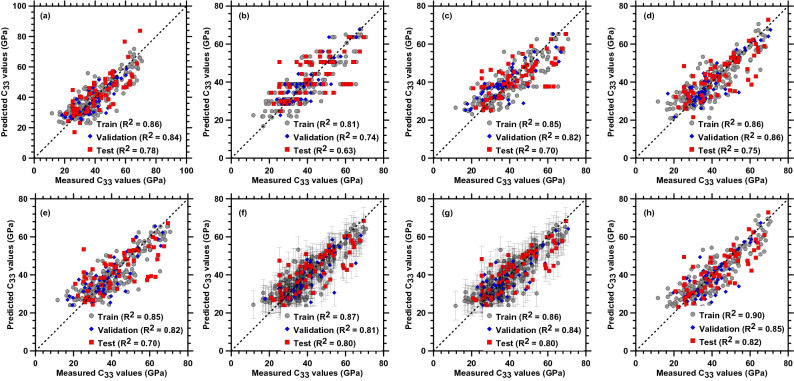
Comparison of the elastic constant C_33_ predicted by (**a**) PR, (**b**) DT, (**c**) RF, (**d**) SVM (with RBF kernel), (**e**) k-NN, (**f**) GPR (with Matern kernel), (**g**) GPR (with RBF kernel) and (**h**) NN with measured values computed by MD simulation. The error bars shown for each value represent the standard deviation around the mean values.

Figure 4 shows the comparison of the elastic constant C_33_ predicted by PR, DT, RF, SVM (with RBF kernel), kNN, GPR (with both Matern and RBF kernels), and NN. From Fig. 4, it is clearly seen that GPR and NN perform the best among all the other models. Among GPR models, the RBF kernel shows better prediction than the Matern kernel. Henceforth, the forthcoming sections focus on GPR with RBF kernel and NN for a detailed evaluation of the constitutive relationships of C–S–H. The results and adopted methods for all other models are sufficiently detailed in the Supplementary document.

### Prediction of elastic constants using Gaussian process (GP)

In this section, predictions based on GP regression (see “Methods” section) are the focus. Two kernels i.e., radial basis function (RBF) and Matern kernels which are commonly adopted in the literature and also have been shown to produce accurate results^38^ are implemented here.

**Figure 5:**
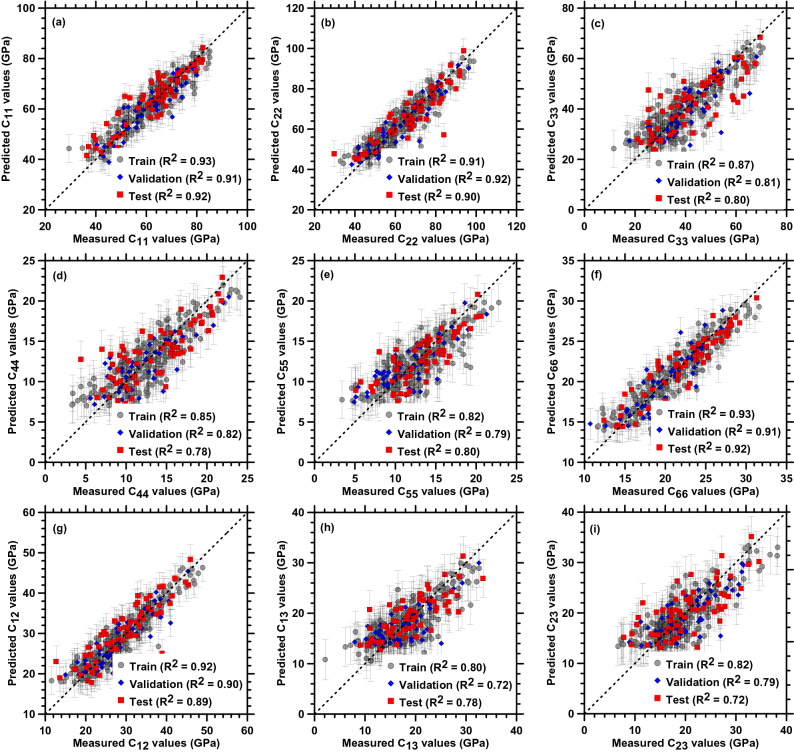
Comparison of the predicted elastic constants by GPR (with RBF kernel) and measured values which are computed by MD simulation. The error bars shown for each value represent the standard deviation around the mean values.

Figure 5 shows the predicted elastic constants using GPR with rbf kernel against the measured values computed by MD simulation.

Here, the GPR model is trained using a train set by employing the rbf kernel along with white noise, and the model is updated till the hyperparameters converged to a global optimum. It is observed that the GPR model could predict for most of the elastic constants with a higher degree of accuracy except for *C*_44_, *C*_13_, and *C*_23_ for which the R^2^ values were relatively lower. The predicted results for the Matern kernel are provided in the Supplementary document. A comparison between the predictions from both kernels (rbf and Matern) reveals that the results are independent of the choice of kernel.

In a later section, the accuracy of the GP models is compared against NN and other traditional models such as polynomial regression, decision trees, and support vector machine. The advantage of GP regression is its ability to provide the uncertainty underlying in the model. The error bars shown for each value represent the standard deviation around the mean values. Thus, GP regression provides confidence in the predictions, which are lacking in other models. Furthermore, the standard deviation of the training sets represents the level of noise present in the data subjected to the training set. On the contrary, the standard deviation in the test sets corresponds to the uncertainty in the model prediction given the distribution of the training data.

### Prediction of elastic constants using neural network (NN)

**Figure 6:**
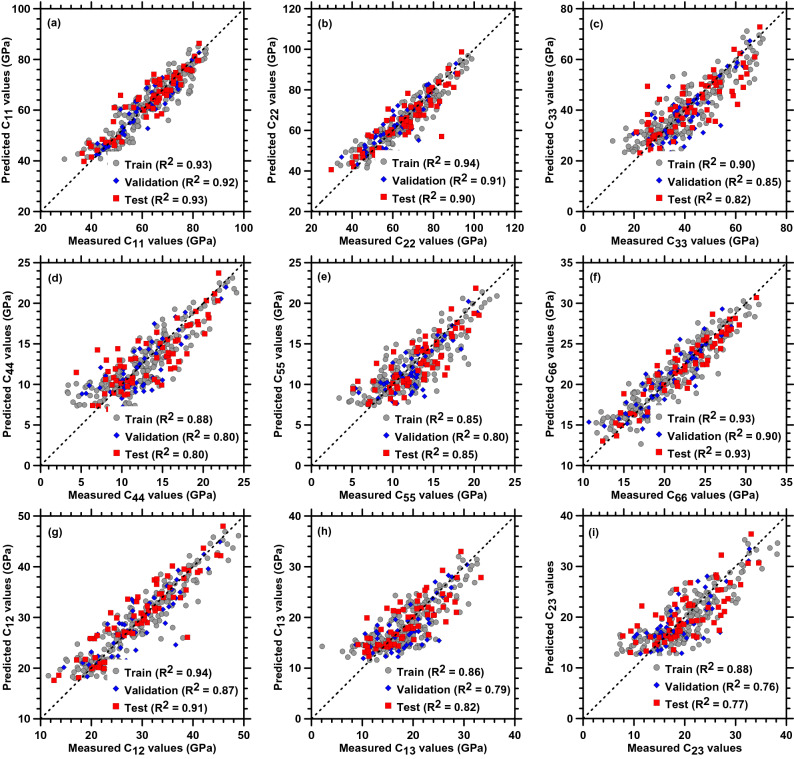
Comparison of the predicted elastic constants from NN (with number of neurons equal to 9) and measured values which are computed by MD simulation.

In this section, the model prediction using NN is assessed. The hyperparameters such as number of hidden layers, number of hidden nodes, optimizer, batch size, number of epochs have been optimized prior to prediction (please refer to Supplementary document). In this NN design, two hidden layers of 9 hidden nodes were used to prevent overfitting of data. With the implementation of NN, MSE for almost all the elastic constants dropped significantly as compared to those observed in the case of other ML techniques. Figure 6 exhibits the predicted responses (using NN with 2 hidden layers and 9 neurons) against the measured values computed by MD simulation. Overall, the prediction accuracy has improved significantly as compared to all other studied models. This is because the neural network implicitly considers all the outputs as dependent, which are overlooked in other models.

## Discussion

### Database adequacy

For adequacy, the database should be (1) balanced, (2) representative, (3) complete, and (4) consistent^39^. In this current study, the dataset is generated by varying the composition of CaO, SiO_2,_ and H_2_O in a uniform fashion. This is to ensure that the data points from all possible regions in the ternary diagram are equally represented. To obtain a representative dataset, the data are split into training (80%) and test set (20%). The hyperparameters are tuned by implementing fivefold cross-validation. At every fold in cross-validation, the training data is further divided into 80% of the training set and 20% for validation. The errors (training error and validation error) calculated from each fold are averaged to represent the average model error. The performance of the model is then evaluated on the unseen test dataset. In this study, a good correlation between the test values and the predicted values is obtained as can be observed from the results presented in Table 2 and Fig. 5. Thus, the dataset can be considered representative. Besides, completeness of the dataset is ensured here by choosing all the possible ranges of the Ca/Si ratio for C–S–H observed experimentally. Moreover, the consistency of the dataset is carefully maintained by following the same C–S–H model construction, molecular loading conditions, and elastic constant evaluation procedure within the high throughput MD simulations for all the C–S–H compositions. Thus, the overall adequacy of the dataset is ensured by careful implementation of all the four above-mentioned criteria during the dataset generation and model training/testing procedure.

### Discussion on comparative performance of different ML techniques

For a direct comparison of different ML techniques used in this study, MSE and R^2^ values (for both train and test set) obtained for different elastic constants are shown in Tables 1 and 2 respectively. The results for PR, RF, DT, kNN, and SVM are detailed in the Supplementary document for ease of reference. While the level of accuracy for the training data infers the interpolation ability of the known data, the level of accuracy for the test data evaluates the prediction ability of the model for unknown data. From all the results using different ML techniques, no direct correlation between MSE and R^2^ was observed. As it is observed that MSE of C_55_ for RF is comparatively low (5.17 GPa^2^ with test set, 1.527 GPa^2^ with train set), but has an R^2^ value of 0.78 was obtained. This signifies that model selection should not solely be based on the high R^2^ value but also should be associated with low MSE value. It is worth mentioning that all these models explained herein except NN consider the outputs to be independent which is likely to impart a significant difference in prediction accuracy for NN as compared to other adopted techniques. Nevertheless, models like GP could still provide good prediction when compared with NN.

**Table 1:** Comparison of mean squared error (MSE) values provided by ML algorithms for the training (value inside the parentheses) and test set.

	PR	RF	DT	kNN	SVM	GP	NN
C_11_	24.341 (26.522)	45.083 (33.192)	56.702 (45.088)	40.772 (22.020)	26.815 (17.885)	25.113 (19.717)	**21.551** (19.381)
C_22_	40.987 (34.132)	55.762 (43.597)	90.555 (57.707)	61.640 (31.085)	53.451 (24.931)	40.671 (30.324)	**39.640** (20.567)
C_33_	68.939 (44.042)	88.791 (14.315)	103.984 (58.083)	90.302 (40.660)	76.873 (41.262)	60.630 (42.460)	**57.241** (38.102)
C_44_	7.675 (4.982)	9.990 (5.823)	11.081 (6.206)	9.384 (4.631)	9.862 (5.276)	7.769 (4.759)	**6.995** (3.647)
C_55_	5.477 (4.712)	5.171 (5.027)	7.501 (6.134)	5.503 (4.463)	5.634 (3.965)	**4.515** (4.702)	4.607 (3.878)
C_66_	2.832 (3.274)	4.361 (4.355)	8.939 (5.904)	4.321 (3.238)	3.829 (2.457)	**3.044** (2.903)	3.176 (2.946)
C_12_	11.816 (11.719)	16.856 (14.399)	20.194 (19.303)	20.816 (10.558)	15.731 (8.900)	12.591 (8.909)	**11.075** (7.063)
C_13_	14.026 (12.880)	17.600 (14.613)	18.099 (15.827)	17.402 (12.007)	15.390 (12.087)	12.368 (12.517)	**11.735** (8.624)
C_23_	19.398 (12.761)	21.549 (13.939)	23.894 (15.834)	25.729 (12.059)	22.549 (11.939)	18.128 (12.475)	**17.704** (8.280)

**Table 2:** Comparison of R^2^ values provided by ML algorithms for the training (value inside the parentheses) and test set.

	PR	RF	DT	kNN	SVM	GP	NN
C_11_	0.911 (0.906)	0.837 (0.876)	0.769 (0.822)	0.851 (0.875)	0.911 (0.936)	0.919 (0.930)	**0.928** (0.930)
C_22_	0.897 (0.911)	0.852 (0.881)	0.771 (0.837)	0.845 (0.881)	0.871 (0.937)	0.901 (0.918)	**0.903** (0.945)
C_33_	0.784 (0.856)	0.695 (0.846)	0.633 (0.811)	0.692 (0.852)	0.748 (0.865)	0.805 (0.866)	**0.817** (0.905)
C_44_	0.790 (0.869)	0.703 (0.827)	0.645 (0.784)	0.702 (0.831)	0.730 (0.879)	0.779 (0.854)	**0.805** (0.888)
C_55_	0.750 (0.846)	0.714 (0.799)	0.685 (0.747)	0.714 (0.804)	0.745 (0.860)	0.802 (0.817)	**0.851** (0.848)
C_66_	0.930 (0.917)	0.861 (0.879)	0.791 (0.839)	0.858 (0.884)	0.907 (0.932)	**0.922** (0.927)	**0.922** (0.922)
C_12_	0.902 (0.895)	0.853 (0.868)	0.763 (0.819)	0.848 (0.865)	0.874 (0.925)	0.898 (0.923)	**0.906** (0.938)
C_13_	0.742 (0.788)	0.687 (0.763)	0.648 (0.730)	0.685 (0.757)	0.734 (0.805)	0.780 (0.800)	**0.819** (0.863)
C_23_	0.723 (0.815)	0.652 (0.799)	0.593 (0.767)	0.651 (0.803)	0.708 (0.842)	0.722 (0.817)	**0.771** (0.883)

From Tables 1 and 2, it is observed that the RF algorithm yield the least MSE and highest R^2^ value for the train set. However, RF suffered from low-level prediction accuracy. A similar observation is also reported for silicate glass in the literature^39^. Results in Tables 1 and 2 also suggest that RF offers better predictability than DT. This is because RF trains a large number of trees individually and its prediction accuracy depends on the decision trees ensemble. On the other hand, the DT algorithm depends on a nodal binary split. Also, in the DT algorithm, based on the selected features and values, the observations are placed to the left node or the daughter node. In the case of the RF algorithm, the output for all the trees is averaged which incorporates non-linearity especially when enough number of trees are used. This is the reason why RF could offer excellent interpolation for the training set but fair prediction of the test set.

It is ideally required for any model to minimize complexity while maintaining high interpretability. However, in general, models that provide higher prediction accuracy often suffer from higher computational complexity and limited or no interpretability. In this study, PR has high interpretability and it is associated with low complexity. Overall, although PR offered good accuracy with lower MSE and fair R^2^ for a train set, however, it falls short when predicting responses using the test set. Besides, the predictability for individual outputs are comparatively low compared to GP and NN. Nevertheless, PR provides us information that the composition–property is not linearly correlated, which is crucial to develop a predictive model.

Lastly, though the model complexity is high for NN as it is associated with two hidden layers and each layer has 9 hidden nodes. Overall, NN by far performed the best in terms of the accuracy for both train and test set for individual outputs. This shows the superiority of the NN for multiple outputs when enough data is trained. One of the drawbacks of NN is that it requires huge computation resources and takes a larger amount of time to train the model.

### Discussion on model interpretation for NN

**Figure 7:**
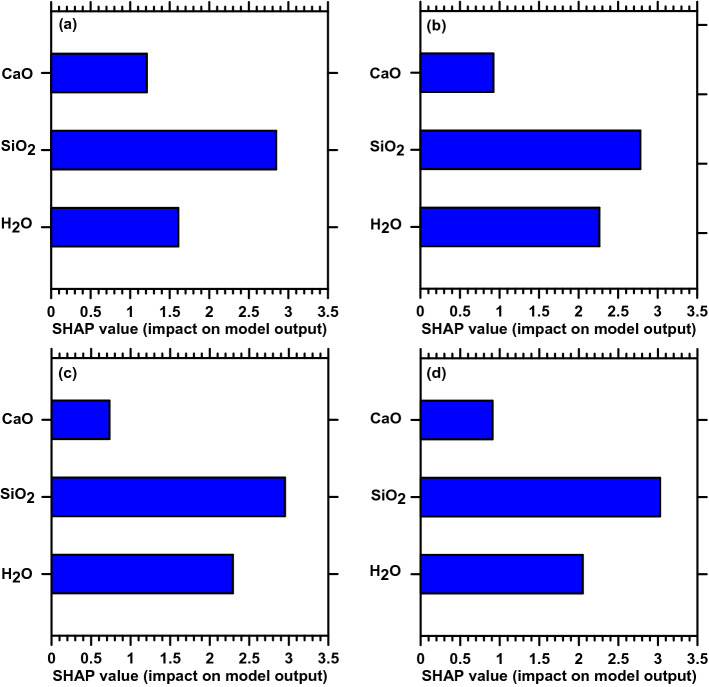
SHAP values for various compositions for (**a**) C_11_, (**b**) C_33_, (**c**) C_44_ and (**d**) C_66_.

This discussion section demonstrates the interpretability of the NN predictions by using SHAP^17^. In SHAP, the impact of each feature on the prediction is obtained by assigning each feature an importance value for a respective prediction. The results are shown in Fig. 7.

The general trend in Fig. 6a–d suggests that all the elastic constants are primarily controlled by SiO_2_ content followed by water and CaO content. While the normal stiffness along the interlayer direction (C_11_) shows a relatively lower SHAP value for water, the value increases when the normal stiffness perpendicular to the interlayer direction (C_33_) is considered. Besides, the shear components (C_44_, C_55_ (please refer to Supplementary Fig. 14), and C_66_ ) show increased contribution from water. This could be attributed to the layered nature of CSH where the in-plane movements are primarily controlled by the water molecules, while the normal stiffness along the direction of the interlayers is mainly controlled by the silicate network (as observed in C_11_ case). Similarly, for other elastic constants such as C_22_, C_55,_ C_12_, C_13_, and C_23_ (see Supplementary Fig. 14) SiO_2_ content primarily dominates followed by water content and CaO content.

## Outlook

This paper establishes that the nature of the input–output relationship of a complex material such as C–S–H can be effectively predicted and interpreted using ML. Due to the limitation of the experimental data available in the literature, especially for different C–S–H compositions, this study uses physics-based MD simulations to generate the elastic constant dataset for different C–S–H compositions. Note that only the compositional ranges of C–S–H that is observed experimentally is used. The molecular structure for each composition is simulated by implementing ReaxFF. Further, instead of a single effective mechanical property such as Young’s modulus or hardness, this study evaluates different individual components of the stiffness moduli, in particular, nine stiffness components. Using the dataset generated from MD simulations, the elastic constants for C–S–H are predicted by implementing two ML techniques: Gaussian Process (GP) and neural network (NN). By judicious selection of optimal level of complexity, and accuracy reliable predictions of the properties can be obtained while ensuring there is no under- or overfitting. A comparative evaluation between the ML techniques reveals that GP and NN show significantly improved predictions as compared to other adopted techniques and NN is found to offer the highest level of accuracy with considerably lower MSE and good R^2^ values.

Furthermore, to interpret the influence of CaO, SiO_2_, and water on various stiffness components of C–S–H, obtained using the NN-based model, SHAP is leveraged which evaluates the importance of each model features on the model’s output after considering all the possible combinations. From evaluations using SHAP, the following conclusions are drawn: (1) all the stiffness components of C–S–H are dominantly influenced by SiO_2_ content followed by water and CaO content; (2) the influence of water content is more prominent for shear components. These results suggest that the in-plane movements are primarily controlled by the water molecules, while the normal stiffness along the direction of the interlayers is mainly controlled by the silicate network. Overall, by synergistically integrating high-throughput MD simulations with ML approaches, this paper shows the efficacy of using ML-based approaches to predict the mechanical behavior of C–S–H and this study can be adopted as a starting point towards developing integrated experiment-multiscale simulation-ML-based design strategies for exceptional materials performance.

## Methods

### High-throughput MD simulations

In this study, high-throughput MD simulations are performed to obtain an adequate dataset of elastic constants for different compositions of C–S–H. The C–S–H model construction procedure for varying Ca/Si ratios, molecular loading conditions, and evaluation of elastic constants for all the C–S–H compositions within the high-throughput MD simulations are presented in the forthcoming sub-sections.

#### C–S–H model construction

Here, the realistic molar percentages of SiO_2_, CaO, and H_2_O are adopted as 11–38%, 23–55%, and 7–66% (molar %) respectively. These ranges are chosen based on viable ranges (Ca/Si molar ratio) of constituents reported in the literature^16,19,20,29^ to form C–S–H. The CSH models are constructed by introducing defects in a layered 11 Å tobermorite^40^ structure. The 11 Å tobermorite configuration contains pseudo-octahedral calcium oxide sheets surrounded by silicate tetrahedral chains, which consists of bridging oxygen (BO) atoms and ${Q}^{2}$ silicon atoms (i.e., Si atom connected to two bridging and two non-bridging terminal oxygen atoms)^41^. Such configuration involves negatively charged calcium-silicate sheets which are separated from each other by interlayer spacings. The interlayer spacing is filled with interlayer water molecules and charged-balancing calcium cations. It is to be noted that the initial configuration of 11 Å tobermorite consists of a Ca/Si ratio equal to 1, this ratio is increased to the range of 1.09–2.06 as constructed in the present models by randomly removing charge-neutral SiO_2_ groups. This removal of SiO_2_ introduces defects in the silicate chains and provides possible sites for adsorption of extra water molecules. To this end the adsorption of water molecules in the structurally defected tobermorite model is performed by implementing the Grand Canonical Monte Carlo (GCMC)^42^ method, ensuring equilibrium with bulk water at constant volume, zero chemical potential, and room temperature. A similar model development procedure for C–S–H has been successfully implemented in the literature^19,21–23,30^ and the procedure is adequately detailed in several published articles^22,30^. ReaxFF is used here in the MD simulations which has been successfully implemented to evaluate the behavior of C–S–H^21,24,30^ and other similar materials^43–45^. These studies have successfully leveraged the features of ReaxFF to evaluate the dynamics of nano-confined water in C–S–H^19^, fracture toughness^24^, structural properties of C–S–H^21,30^, and radiation damage in C–S–H^46^. Besides, ReaxFF potential has been shown to model C–S–H^47^ reliably in terms of the structural and elastic properties as it is based on the bond-formation/breakages, which is useful for reactive mechanisms such as dissociation of nano-confined water in C–S–H. The generated structure is further relaxed at 300 K and zero pressure for 500 ps in the *NVT* and *NPT* ensemble with a timestep of 0.25 fs before computing the stiffness components. The molar range of Ca/Si ratio is maintained consistent with those from the literature^16,29,48^. To obtain different water content, water molecules are randomly removed from the saturated structure and equilibrated for 500 ps in *NVT* and *NPT*, respectively. All the simulations are performed in an open source code LAMMPS package^49^. The methodology for model construction for C–S–H is adequately detailed in the literature^19,22,30^.

#### Molecular loading conditions

Once the structures are adequately equilibrated, they are subjected to three axial and three shear deformations along the X, Y, and Z axes. To apply axial tensile load, the C–S–H structures are subjected to uniform tensile strain in the X-direction, and the process is continued for Y and Z-directions. Similarly, to simulate the shear loading in the C–S–H structures, a shear strain is applied along X, Y, and Z-directions, respectively.

#### Evaluation of elastic constants

During the deformations the elastic constants $C_{ij}$ matrix is obtained as^39^:

1 $$ C_{ij} = \frac{1}{V}\frac{{\partial^{2} U}}{{\partial \epsilon_{i} \epsilon_{j} }} $$

where $U$ is the potential energy, $V$ is the volume of the structure, $\epsilon$ is the strain, i and j are the indexes representing each Cartesian direction. In this study, 9 components of stiffness moduli are considered for prediction ($C_{11} , C_{22} , C_{33} , C_{44} , C_{55} , C_{66} , C_{12} , C_{13}$ and $C_{23}$). The same has been adopted when calculating the elastic properties such as Young’s modulus, shear modulus, and bulk modulus from the stiffness matrix in glass structure using MD simulation^50,51^.

All the simulations are conducted using the Large-scale Atomic/Molecular Massively Parallel Simulator (LAMMPS) package^49^. Each C–S–H structure comprises at least 3000 atoms. ReaxFF is used as an interatomic potential. The process is repeated till all the elastic constants for different Ca/Si ratio with different water has been generated”.

### Machine learning (ML) techniques

The database of the stiffness matrix is computed from the MD simulations to predict composition-dependent elastic constants for C–S–H using various ML techniques. This paper primarily focuses on Gaussian process (GP), and neural network (NN) which are discussed in the forthcoming sub-sections. Besides, this paper also evaluates other common ML techniques such as polynomial regression, random forest, support vector machine, k-nearest neighbors, and decision trees for comparative assessment of prediction abilities. These common ML techniques are detailed in the Supplementary document for ease of reference.

#### Gaussian processes regression

A Gaussian process is defined as a collection of random variables among which a finite subset has a joint Gaussian distribution^52^. One can implement it to describe a distribution over a given set of input(x) and output datasets (y). For a linear regression model with noise $\epsilon$,

2 $$ y = f\left( x \right) + \epsilon ; \epsilon \sim {\mathcal{N}}\left( {0,\sigma_{\epsilon }^{2} } \right) $$

where the noise is assumed to follow an independent, identically distributed Gaussian distribution with zero mean and variance ($\sigma_{\epsilon }^{2}$). Without losing generality, a Gaussian process can be completely described by its mean function and covariance function,

3 $$ f\left( x \right)\sim {\mathcal{G}\mathcal{P}}\left( {m\left( x \right),k\left( {x,x^{\prime}} \right)} \right) $$

where ${\mathcal{G}\mathcal{P}}\left( \cdot \right)$ is the specified Gaussian process, $m\left( x \right)$ is the mean function which computed the expected values of output for a given input, and $k\left( {x,x^{\prime } } \right)$ is the covariance function, a Gaussian prior function that captures the extent of correlation between function outputs for the given sets of inputs. The covariance function is expressed as:

4 $$ k\left( {x,x^{\prime } } \right) = E\left[ {f\left( x \right) - m\left( x \right), f\left( {x^{\prime } } \right) - m\left( {x^{\prime } } \right)} \right] $$

Instead of using a specified functional form (as in the case of deterministic model), Gaussian processes describe the input–output relationship through distributions over functions of the input space, $x \in {\mathcal{X}}$. The designated random variables follow Gaussian distribution. For Gaussian distribution, the marginalization and conditioning properties can be fully utilized to obtain the marginal likelihood and the conditional probability via the designated mean and covariance. For the mean-subtracted data set, the mean function is set to zero and the prior’s covariance is specified by assigning trial kernel functions with a set of hyperparameters. The widely used kernel functions are exponential kernel and squared exponential kernel. To obtain the posterior distribution over functions, one must restrict the joint prior to containing only those functions which agree with the training data through conditioning of the Gaussian prior. The joint distribution of the training outputs, y, and the test outputs $f^{\prime}$ according to the prior is expressed as^52^:

5 $$ \left( {\begin{array}{*{20}c} y \\ {f^{\prime } } \\ \end{array} } \right)\sim {\mathcal{N}}\left( {\begin{array}{*{20}c} {0,} & {\begin{array}{*{20}c} {K\left( {X,X} \right) + \sigma_{\epsilon }^{2} I} & {K\left( {X,X^{\prime } } \right)} \\ {K\left( {X^{\prime } ,X} \right)} & {K\left( {X^{\prime } ,X^{\prime } } \right)} \\ \end{array} } \\ \end{array} } \right) $$

If there are $n$ training points and $n^{\prime }$ testing points then $K\left( {X,X} \right)$ is a $n \times n$ matrix of the covariance between all observed training points, $K\left( {X,X^{\prime } } \right)$ is the $n \times n^{\prime }$ covariance matrix between the training and testing pairs and likewise for $K\left( {X^{\prime } ,X} \right)$ and $K\left( {X^{\prime } ,X^{\prime } } \right)$. Applying principles of conditionals, the marginal likelihood of the output can be assumed to follow a gaussian distribution with the predicted mean $m\left( {f^{\prime } } \right)$ and covariance function $k\left( {f^{\prime } } \right)$ as^52^:

6 $$ f^{\prime } |X,y,X^{\prime } \sim {\mathcal{N}}\left( {m\left( {f^{\prime } } \right),k\left( {f^{\prime } } \right)} \right) $$

7 $$ m\left( {f^{\prime } } \right) = K\left( {X^{\prime } ,X} \right)\left( {K\left( {X,X} \right) + \sigma_{\epsilon }^{2} I} \right)^{ - 1} y $$

8 $$ k\left( {f^{\prime } } \right) = K\left( {X^{\prime } ,X^{\prime } } \right) - K\left( {X^{\prime } ,X} \right)\left( {K\left( {X,X} \right) + \sigma_{\epsilon }^{2} I} \right)^{ - 1} K\left( {X,X^{\prime } } \right) $$

The marginal likelihood of the output given the input can be obtained through the marginalization and the model is selected by updating the hyperparameters during training through the maximization of the marginal-likelihood (or log-marginal-likelihood). The set of hyperparameters should ideally converge to a global optimum.

#### Neural network (NN)

Neural network is a mathematical model which maps a given set of predictors, $x$, to a set of desired response, $y$. The early proposition of this idea is linked to the assumption of how the information is stored and processed in the brain^53^. The map between the predictor and the response is comprised of multiple layers of perceptron and activation functions and it is called the feed-forward neural network. The estimated response can be expressed as follows,

9 $$ {\varvec{y}} = {\varvec{f}}_{{\varvec{N}}} \left( {{\varvec{A}}_{{\varvec{N}}} , \ldots {\varvec{f}}_{2} \left( {{\varvec{A}}_{2} ,{\varvec{f}}_{1} \left( {{\varvec{A}}_{1} ,{\varvec{x}}} \right)} \right) \ldots } \right) $$

where ${\varvec{f}}_{{\varvec{N}}} \left( \cdot \right):{\mathbb{R}} \to {\mathbb{R}}$ is a continuous bounded function which is usually referred to as the activation function, ${\varvec{A}}_{{\varvec{i}}} :{\mathbb{R}}^{{{\varvec{d}}_{{\varvec{i}}} }} \to {\mathbb{R}}^{{{\varvec{d}}_{{{\varvec{i}} + 1}} }}$ is the transformation matrix that contains weights between two layers of perceptron^54^. The neural network received very much attention in academia and applications in engineering due to the proven universal approximation property which states that the feed-forward neural network architectures with a sigmoid activation function are capable of approximating any set of functions between two Euclidean spaces for the canonical topology^55^.

The weights can be solved by formulating the above mapping into a constrained optimization problem as stated below,

10 $$ {\varvec{argmin}}_{{{\varvec{A}}_{{\varvec{j}}} }} \left\{ {f_{{\varvec{N}}} \left( {{\varvec{A}}_{N} , \ldots {\varvec{f}}_{2} \left( {{\varvec{A}}_{2} ,\left( {{\varvec{f}}_{1} \left( {{\varvec{A}}_{1} ,{\varvec{x}}} \right)} \right) \ldots } \right) + {\mathbf{\lambda g}}\left( {{\mathbf{A}}_{{\mathbf{j}}} } \right)} \right)} \right\} $$

where λ is the regularization intensity constant and $g\left( \cdot \right)$ is a functional form of the weights to be regularized. This optimization problem is usually solved by stochastic gradient descent or backward propagation algorithm. Since the non-convex nature of the neural network, the solution to this optimization problem is not unique. Moreover, the selection of the number of layers and the number of perceptron in each layer affects the result of the regression, and it is subjected to high variance problems when large numbers of neurons and layers are used. As such proper regularization is needed when the neural network is implemented. In this study, while training a neural network model, a rectified linear unit (ReLU) is implemented for performance-enhancement. Here, the data is trained using a feedforward multilayer perceptron where the weights are trained by the back propagation algorithm. Henceforth, the feedforward backpropagation multilayer perceptrons will be referred to as a neural network (NN), which is commonly used in the literature.

### Model tuning and cross-validation

To avoid the possibility of overfitting the data, 20% of the data is set aside from the models for its intended use as a “test set” to assess the performance of the ML algorithms on these unseen data. To this end, a *k*-fold cross-validation (CV) technique is adopted in this study. In the CV technique, the dataset is split into *k* number of smaller sets, where in each fold the model is trained on a fraction of data (train set) and tested on the remaining data. The final value obtained is the average value which is iteratively run on each of the *k*-folds. To this end, this study adopts a nested two-level CV approach as detailed in the article by Cawley and Talbot^56^. First, the dataset is split into the training set (which is 80% of the data) and test set (20% of the data). In outer CV the model is run for the number of iterations and the average value of the scores (i.e. $R^{2}$ and MSE) obtained from each fold is used to obtain a comparative performance-evaluation of various ML techniques. In order to obtain the appropriate hyperparameters, a fivefold inner CV is implemented for the training dataset. This nested CV technique alleviates some of the issues regarding the limitations of relatively smaller datasets.

It is challenging in ML to obtain a model that is accurate and simple at the same time. Simplistic models show a lower degree of prediction accuracy or are under fitted, whereas overly complex models often performed worst on the test data or unknown sets of data. Such models can capture perfect trends on the training dataset but show poor transferability to unknown sets of data and suffer from overfitting. Hence, models need to be optimized by tuning the hyperparameters so that an ideal trade-off between accuracy and computational demand is reached.

### Model evaluation metrics: mean square error (MSE)

The mean square error measures the average Euclidean distance between the predicted and true or measured values and is expressed as:

11 $$ MSE = \frac{1}{n}\mathop \sum \limits_{i = 1}^{n} \left( {y_{p} \left( i \right) - y_{t} \left( i \right)} \right)\left( {y_{p} \left( i \right) - y_{t} \left( i \right)} \right) $$

where $y_{t} \left( i \right)$ is the *i*th true output and $y_{p} \left( i \right)$ is the *i*th predicted output. MSE serves as an indicator of prediction accuracy and MSE needs to be minimized in order to maximize the accuracy of ML algorithms.

### Model evaluation metrics: Linear coefficient of determination, ***R***^2^

In this regression problem, the MSE is majorly selected for the quantification of the model performance on the given data set. The coefficient of determination can be used to quantify the proportion of the variance in the dependent variable that is predictable from the independent variable. In this study, to further assist the model selection in this multiple-input multiple-output (MIMO) regression problem, the Pearson correlation coefficient^57^ is used to indicate the accuracy of the predicted results.

In the case of the sampled data, the Pearson correlation coefficient can be determined as follows:

12 $$ R^{2} = \frac{{\mathop \sum \nolimits_{i = 1}^{n} \left( {y_{p} \left( i \right) - \overline{y}_{p} } \right)\left( {y_{t} \left( i \right) - \overline{y}_{t} } \right)}}{{\sqrt {\mathop \sum \nolimits_{i = 1}^{n} \left( {y_{p} \left( i \right) - \overline{y}_{p} } \right)^{2} } \sqrt {\mathop \sum \nolimits_{i = 1}^{n} \left( {y_{t} \left( i \right) - \overline{y}_{t} } \right)^{2} } }} $$

Here $y_{t} \left( i \right)$ is the *i*th true output and $y_{p} \left( i \right)$ is the *i*th predicted output.

In this study, both *MSE* and *R*^2^ of the train and test data are used to evaluate the performance of ML algorithms.

### Training process and model refinement

This section describes the training and model fitting (overfitting, underfitting, or balanced) for all the adopted methods. The total data is initially split into a training set and test set by 80:20 proportion. While the test dataset is kept unseen during the model training process, the training set is further split into 80% for training and 20% for validation. Here, to validate the model, a fivefold cross-validation is implemented. The optimum complexity is achieved when the minimum error for both the training error and validation error is achieved.

For PR, the complexity is increased with respect to polynomial order from 1 to 6, and an optimum polynomial order of 3 is obtained (please refer to Supplementary Fig. 1). For SVM with RBF kernel, two parameters are considered, which are C and gamma ($\gamma$). The model complexity is varied by varying C from 0.001 to 1000 and gamma from 0.1 to 10. The parameters are optimized using a grid search approach so as to minimize the error. Thus, optimum values of 100 and 0.46 are obtained for C and $\gamma$ respectively (please refer to Supplementary Fig. 4). In the k-nearest neighbor method, the k-value is varied from 1 to 9 and an optimum value of 4 is achieved (please refer to Supplementary Fig. 6). In the decision tree algorithm, the model complexity is characterized by the maximum tree depth which is varied from 2–10. By evaluating the MSE and the R^2^ values, an optimum value of 5 for the maximum tree depth is chosen (please refer to Supplementary Fig. 8 for more details). For the random forest algorithm, the model complexity is varied by varying the number of trees from 2 to 10 from which an optimal number of 9 for the number of trees is selected which shows the least error for the validation dataset (please refer to Supplementary Fig. 10 for more details). For the Gaussian process, two covariance functions (RBF and Matern) with noise are implemented and the parameters are converged when the log marginal likelihood is maximized. Lastly, for NN the hyperparameters include the number of hidden nodes, size of hidden layers, optimizer function, learning rate, epoch, and batch size. In this study, the Adam optimizer is implemented. The learning is optimized for learning rate equal to 10^–3^, epoch = 400, batch size of 32, and two hidden layers with a number of hidden nodes (or neurons) equal to 9 (please refer to sSpplementary Fig. 13 for more details). Overall, a rigorous hyperparametric optimization methodology employing a grid search was used for model refinement, thereby, ensuring the optimality of the model without underfitting or overfitting. To evaluate the performance of each model, the models are tested using the unseen test dataset. The performance of various methods is evaluated by comparing the MSE and R^2^ values obtained from each model.

### Model interpretability

The ability of the ML techniques such as NN to predict the target accurately by learning from data has been remarkable. However, because of the higher model complexity for algorithms such as NN, the model interpretability becomes challenging. Several studies have tried to address this issue by measuring a few specific features that are responsible for a model’s output^58^. Recently, Shapley Additive Explanations (SHAP) which is derived from Shapley values in game theory^59^ is employed to measure the importance of various features within the model^17,18^. SHAP has been used for various applications across a wide range of disciplines which includes identification of patient risk factors in tree-based medical diagnostic models^60^ and determination of various important features of satellite images which are crucial in generating poverty maps^61^. As per SHAP, the importance of feature $j$ for the output of model $f$, $\phi^{j} \left( f \right)$, is a weighted sum of the feature’s contribution of the model’s output $f\left( {x_{i} } \right)$ over all possible feature combinations^62^. $\phi^{j} \left( f \right)$ is expressed as:

13 $$ \phi^{j} \left( f \right) = \mathop \sum \limits_{{S \sqsubseteq \left\{ {x^{1} , \ldots ,x^{p} } \right\}\backslash \left\{ {x^{j} } \right\}}} \frac{{\left| S \right|!\left( {p - \left| S \right| - 1} \right)!}}{p!}\left( {f\left( {S \sqcup \left\{ {x^{j} } \right\}} \right) - f\left( S \right)} \right) $$

where $x^{j}$ is feature $j$, $S$ is a subset of features, and $p$ is the number of features in the model.

## Supplementary information

Supplementary Information.

## Data Availability

The data that support the findings of this study are available from the corresponding authors upon reasonable request.
