# Supplementary material for: Elucidating the constitutive relationship of calcium–silicate–hydrate gel using high throughput reactive molecular simulations and machine learning
Source: Sci Rep. 2020 Dec 7;10:21336. doi: 10.1038/s41598-020-78368-1 (PMC7721899; doi:10.1038/s41598-020-78368-1)
Supplement: Supplementary file 1 — Supplementary Information. [file 41598_2020_78368_MOESM1_ESM.pdf]

**Supplementary information to**

# Elucidating the Constitutive Relationship of Calcium–Silicate–Hydrate Gel using High Throughput Reactive Molecular Simulations and Machine Learning

Gideon A. Lyngdoh[1], Hewenxuan Li[2], Mohd Zaki[3], N.M. Anoop Krishnan[3,4,*], and Sumanta Das[1,*]

[1]Department of Civil and Environmental Engineering, University of Rhode Island, Kingston, RI, USA

[2]Department of Mechanical, Industrial and Systems Engineering, University of Rhode Island, Kingston, RI, USA

[3]Department of Civil Engineering, Indian Institute of Technology Delhi, Hauz Khas, New Delhi, 110016, India

[4]Department of Materials Science and Engineering, Indian Institute of Technology Delhi, Hauz Khas, New Delhi, 110016, India

[*]Corresponding authors: N. M. A. Krishnan (krishnan@iitd.ac.in), S. Das (sumanta_das@uri.edu)

## PREDICTION OF CONSTITUTIVE BEHAVIOR USING COMMON MACHINE LEARNING TECHNIQUES

### Polynomial Regression (PR)

Polynomial regression is a generalization of linear regression in which the selected predictors are mapped to a higher dimensional feature space according to the desired polynomial order. Polynomial regression is relatively easier to interpret when the polynomial order is low, indicating a lower-dimensional correlation between the dependent variable(s) and the mapped feature coordinates. In general, polynomial regression with $N^{th}$ degree can be expressed as:

$$y = \beta_0 + \sum_i^N \beta_i x^i \quad [1]$$

where $x^i$ is the input variable (or predictor variable) and y is the output (or response) variable. The terms $\beta_0$ and $\beta_i$ are the fitting parameters corresponding to each degree $i$. In matrix form, the formulation can be separated into two phases. First, the vector of predictors is mapped to higher polynomial dimensions,

i.e., $\boldsymbol{x}^i, i = 2, 3, \dots, n$. Second, the mapped higher-order polynomial predictors are used to formulate a regression problem identical to the linear regression,

$$\boldsymbol{y} = \boldsymbol{\beta X} + \beta_0 \quad [2]$$

Using the least-square method, the coefficients ($\beta_0$ and $\beta$) can be estimated by minimizing the error, which is the sum of the squared difference between the true responses with those predicted responses. Hence, the complexity of the PR models highly depends on the choice of the $N^{th}$ polynomial degree considered. After obtaining $\beta_0$ and $\beta$, the unknown variable vector can be obtained from the new predictor vectors, $\boldsymbol{X_p}$ as follows:

$$\hat{\boldsymbol{y}} = \boldsymbol{\beta X_p} + \beta_0 \quad [3]$$

The linear model with polynomial mapped features is selected by comparing the MSE with increasing polynomial order of the features. Without losing generality, the polynomial features are not limited to only the crossing terms. Supplementary Figure 1 (a) and (b) plots the mean MSE and $R^2$ respectively considering all nine elastic constants. As one can observe from Supplementary Figure 1(a), the MSE for both train and validation set decreases with an increase in polynomial order up to a polynomial order of 3 beyond which the MSE for validation set starts to increase whereas the train set shows relatively less variations in MSE. To further complement the accuracy of the models, the corresponding $R^2$ is given in Supplementary Figure 1(b), which shows relatively constant $R^2$ for the train set and a significant decrease in $R^2$ for the validation set with an increase in polynomial order.

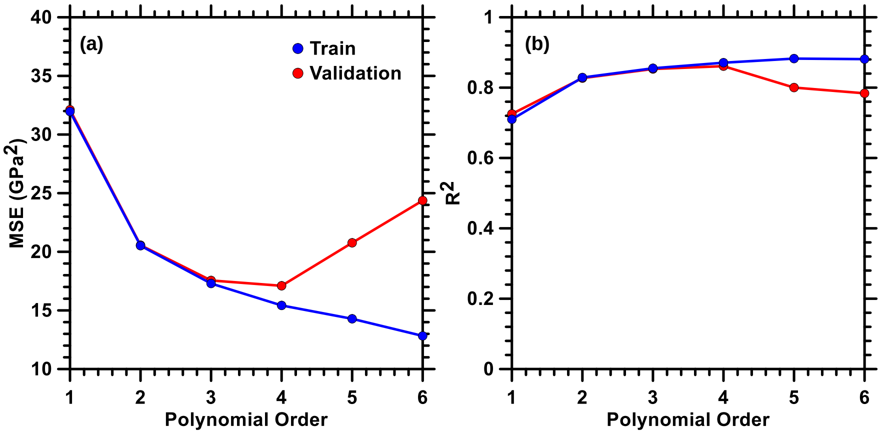


***Supplementary Figure 1***. *(a) MSE and (b) $R^2$ values of the polynomial regression models as a function the maximum polynomial order considered in each model as obtained for the train and validation set*

Such trends beyond a polynomial order of 3 imply overfitting. To shed more light on this, Supplementary Figure 2 plots MSE for all the elastic constants individually as functions of polynomial order.

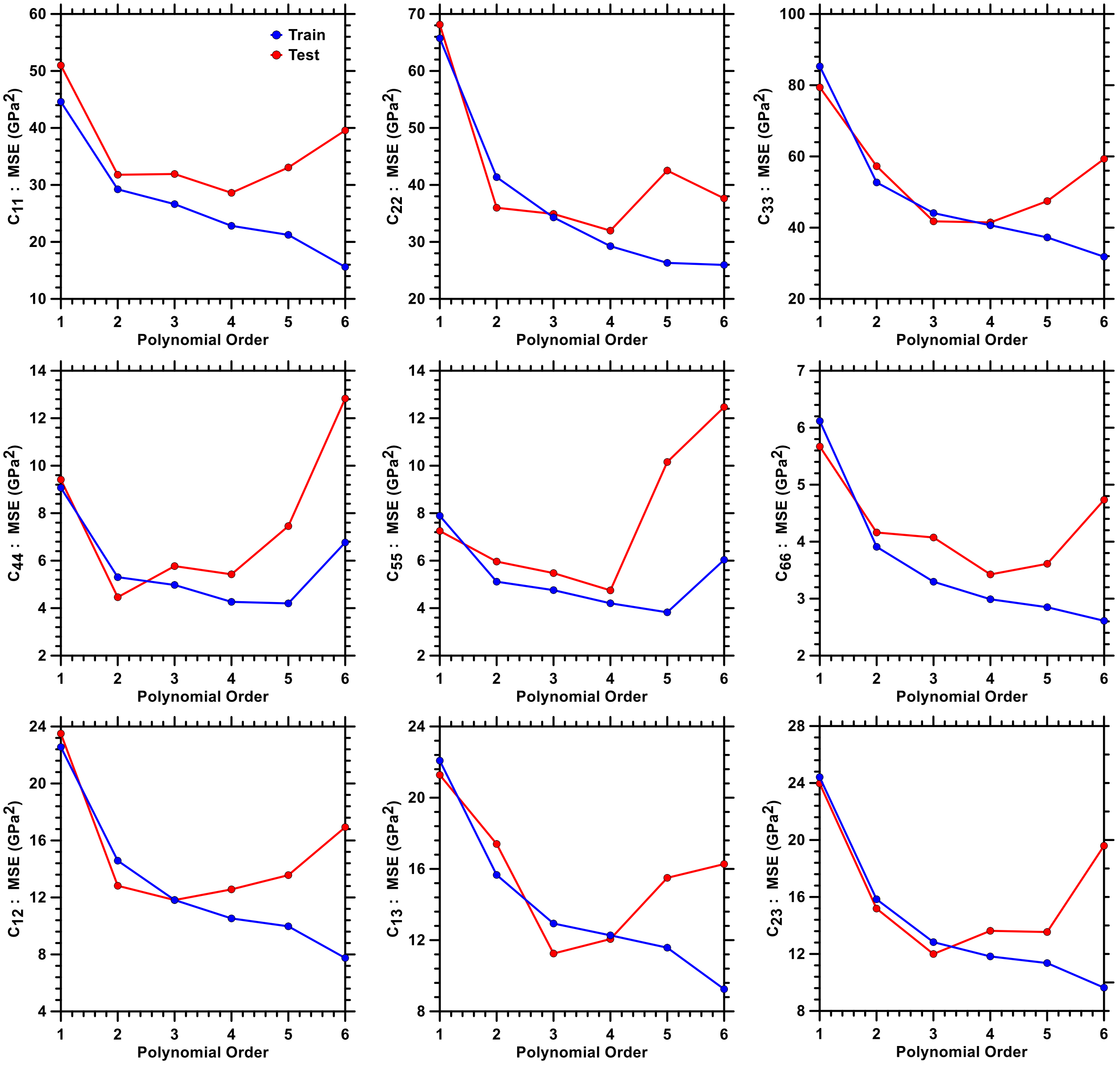


***Supplementary Figure 2.*** *Comparison of the predicted stiffness components by polynomial regression (with a degree equal to 3) and measured value which is computed by molecular dynamics simulation.*

Beyond a polynomial order of 3, almost all the cases show a significant increase in MSE for validation set with an increase in polynomial order. The train set also shows an irregular trend beyond a polynomial order of 3 (for the case of $C_{44}$ and $C_{55}$). The overall trend (the validation errors deviate farther from the train errors with increase in polynomial order beyond 3) from Supplementary Figure 2 indicates significant

overfitting effects beyond the polynomial order of 3 (except for the case of $C_{44}$ and $C_{55}$). As such, an ideal polynomial order of 3 is chosen here.

The prediction results are shown in the Supplementary Figure 3 by directly overlaying the prediction over the train sets and the validation sets for all nine components of the elastic modulus.

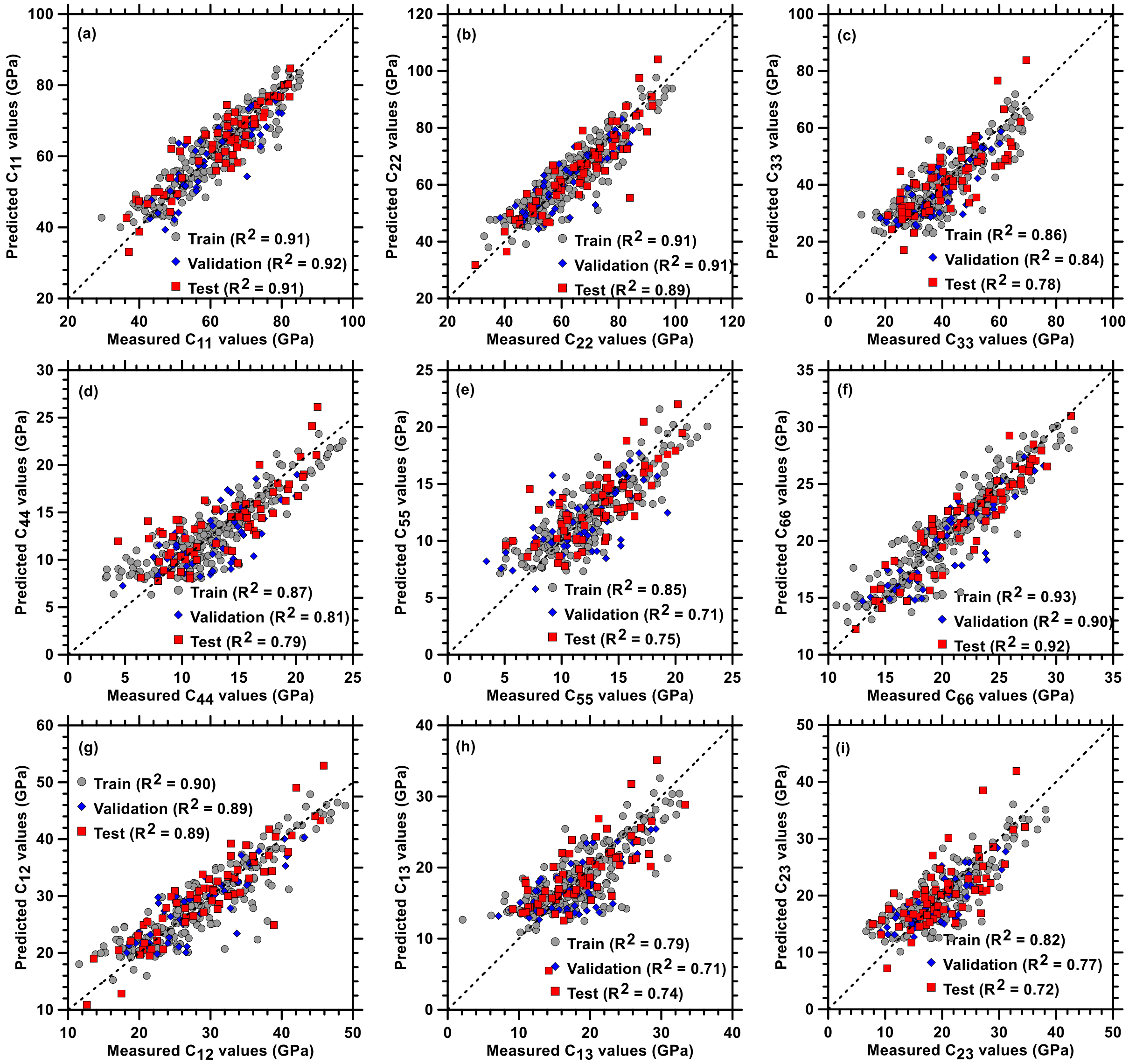


***Supplementary Figure 3.*** *Comparison of the predicted stiffness components by polynomial regression (with a degree equal to 3) and measured value which is computed by molecular dynamics simulation.*

While for most of the cases higher $R^2$ values are obtained, a few cases (such as $C_{13}$, $C_{55}$, $C_{23}$) show relatively lower $R^2$ values due to the presence of irregularities in the C-S-H structure in the form of interlayer spaces.

Nevertheless, the overall results suggest that reasonable prediction accuracies ($R^2$>0.7) can be achieved for all the elastic constants of C-S-H.

**Support Vector Machine (SVM)**

The support vector machine is a support vector classifier that determines the best separating hyperplanes in a higher-dimensional space of the original space of the predictors[1]. The realization of raising the predictors to a space of higher dimension is based on the kernel tricks applied to the predictors. The support vector regression is a convex optimization problem which gives a unique solution to a given set of predictors and responses. The support vector regression can be expressed as follows,

$$\underset{\beta,\beta_0}{argmin}\left\{1/2\left|\left|\beta\right|\right|^2 + C\sum\left|\xi_i\right|\right\} \text{ subject to } |y_i - \beta^\intercal x_i - \beta_0| \leq \epsilon + |\xi_i| \quad [4]$$

Where $\epsilon$ is the pre-defined margin size or the maximum error tolerated by the model, $\xi_i$ is the slack variable which accounts for the tolerance of out-of-margin data points, and $C$ is the constraint of overall tolerance of the out-of-margin cases for finding the SVM model. This constraint acts as a regularization term. As $C$ increases, the regression result is less prone to overfitting the given data. In this study, a radial basis function (RBF) kernel is adopted.

Supplementary Figure 4(a) and (b) show the plot of MSE and $R^2$ of the SVM model (with RBF kernel) with increasing gamma ($\gamma$) value.

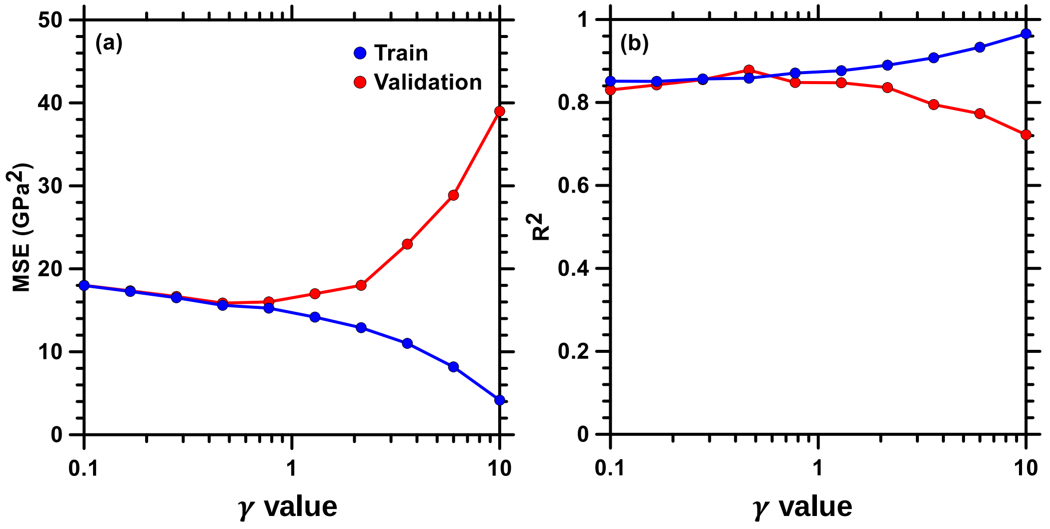


***Supplementary Figure 4**. (a) MSE value of the support vector machine models (with RBF kernel) as a function of gamma value considered in each model as obtained for the train and validation set. (b) $R^2$ of the train set and validation set with respect to the gamma value considered in each model.*

It is observed that with a low gamma value, the model is underfitting and the error value is comparatively higher for both train and validation set. On the contrary, with high gamma value, the MSE of the validation set starts increasing, and for $R^2$ starts decreasing as shown in Supplementary Figure 4(a) and (b). An optimum gamma value of 0.46 is chosen where minimum MSE and high $R^2$ for validation set is achieved. The C value adopted in this model is 100. Using the optimal gamma value of 0.46, the prediction results are plotted against measured values from MD simulations in Supplementary Figure 5 for all the elastic constants.

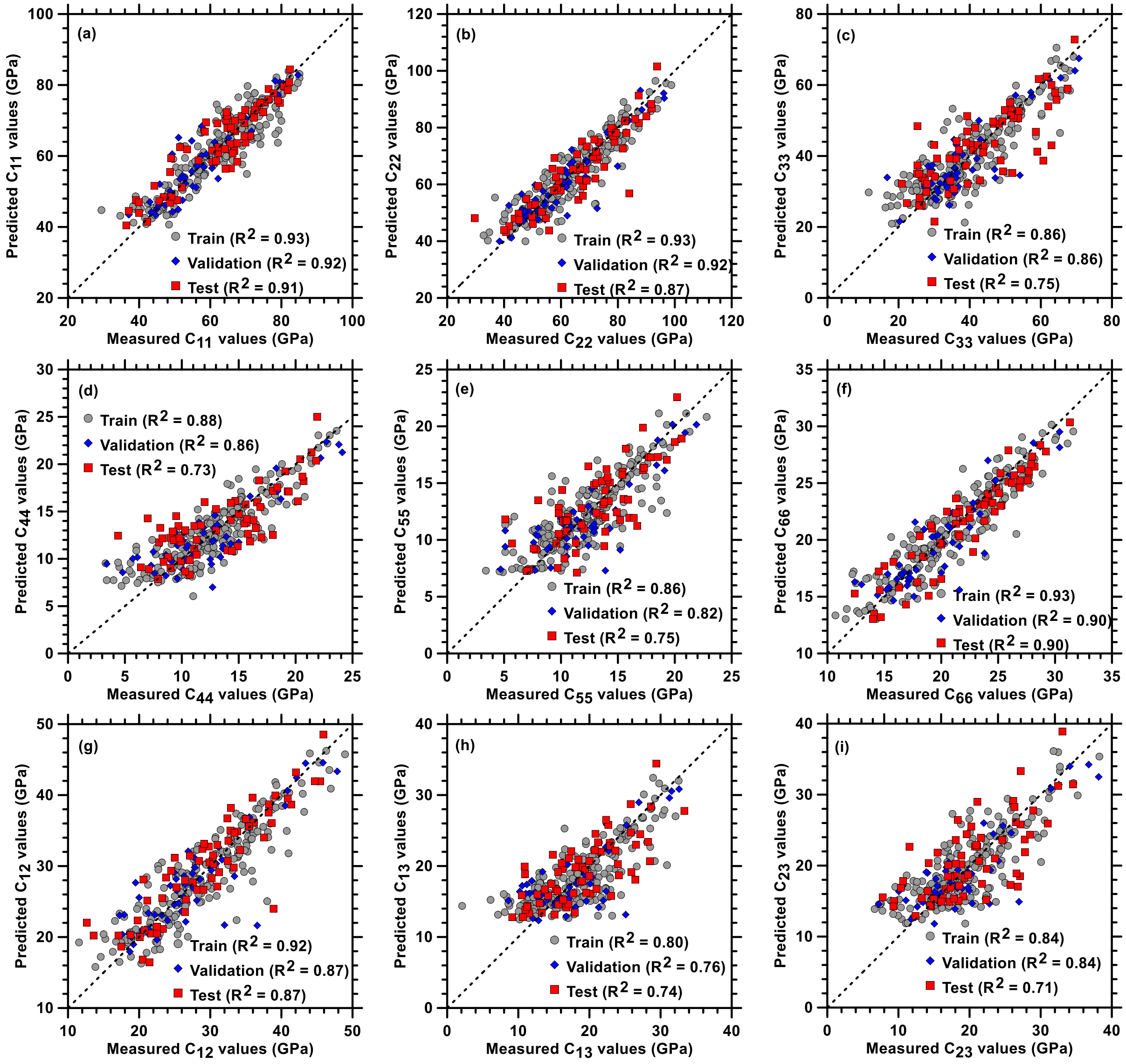


***Supplementary Figure 5.*** *Comparison of the predicted stiffness components by SVM (with gamma value equal to 0.46) and measured values which are computed by molecular dynamics simulation.*

The $R^2$ value for different outputs are also computed. While the algorithm performs well for $C_{11}$, $C_{22}$, $C_{66,}$ and $C_{12}$, some of the components (such as $C_{13}$) show relatively lower $R^2$ values for validation set.

**k-Nearest Neighbor (kNN)**

kNN is a simple ML technique that is used for both classification and regression problems. In kNN, the predictions are based on the entire train dataset by calculating the similarity between the input sample and each training instance. It is also a non-parametric model as it does not make strong assumptions about the internal mapping function and this is added to the model to be more flexible as it has the freedom to learn any functions from the training dataset. Specifically, KNN regression is implemented based on the K nearest neighbors for each query point, and K is a model hyperparameter defined by the user. There are generally two modes in terms of calculating contributions from each neighbor of the query point. These are equal weight contribution, and inverse distance weight contribution. In this work, we have used the conventional equal weight contribution mode.

Supplementary Figure 6(a) and (b) show the plot of MSE and $R^2$ obtained from k-nearest neighbors (considering mean error for all the nine elastic constants) with an increase in k-value.

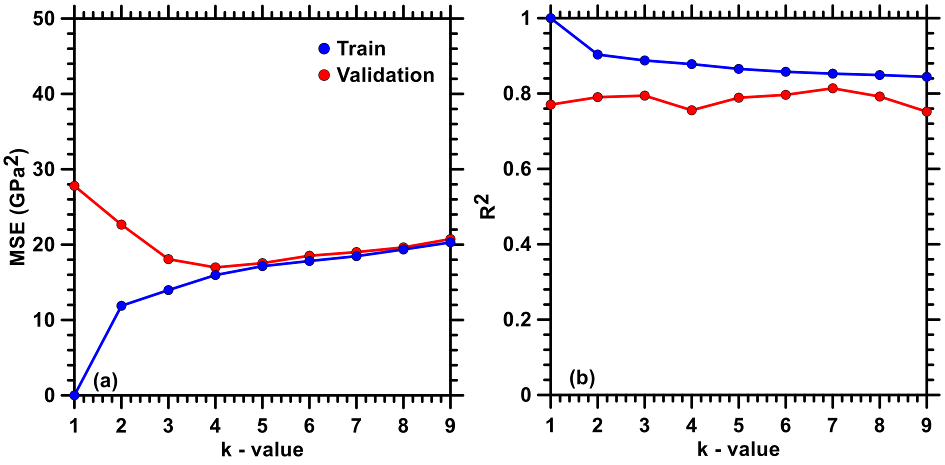


***Supplementary Figure 6***. *(a) MSE and (b) $R^2$ values of the k-nearest neighbor models as a function of k-value considered in each model as obtained for the train and validation set. The optimal order is chosen for such a model where minimum MSE and maximum $R^2$ of the validation set are observed.*

Here, the k-value characterizes the complexity of the model. For a very low value of k (equal to 1), the model tends to overfit for the train set which leads to high error values when tested with the validation set. On the other hand, for a high value of k, the model performs poorly on both the train and validation set. It can be clearly seen from the Supplementary Figure 6(a) that the validation error reaches minima

and $R^2$ value reaches its peak at a value of k equals to 4. Any further increase in k values does not improve the prediction accuracy. Thus, a k-value equals to 4 can be taken as the optimum value for the model.

Supplementary Figure 7 shows the predicted response vs measured values from MD simulations for all the elastic constants with the corresponding $R^2$ values. Overall, the k-Nearest Neighbor algorithm shows good prediction accuracy for $C_{11}$, $C_{22}$, $C_{12}$, and $C_{66}$ whereas the $R^2$ values are found to be relatively lower for the others.

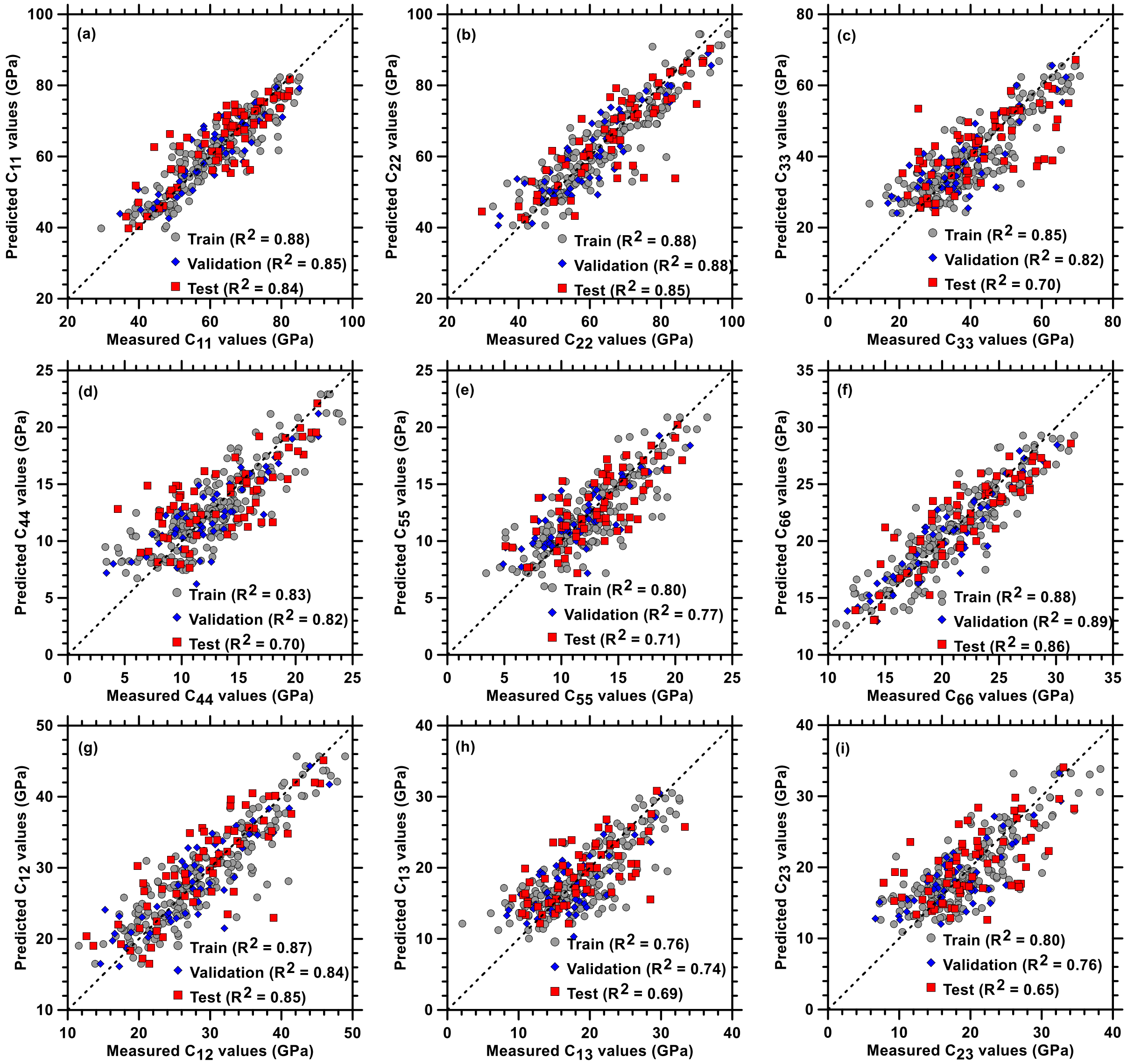


***Supplementary Figure 7.*** *Comparison of the predicted stiffness components by kNN (with k = 4) and measured values which are computed by molecular dynamics simulation.*

**Decision tree**

Decision trees are a family of ML techniques that are used for both classification and regression problems [2–4]. While the other regression methods such as neural network fit a set of parameters in a mathematical formula, decision trees are "rule-based" models where they aim to identify logical splits in the data [2]. In this algorithm, the input space is split into a series of partitions (also denoted as leaf nodes), and then a simple model (i.e., often simply a constant value) is used to predict the output in each leaf node [2]. The splits are selected such that a minimum value of error is obtained. A popular method to determine the splits is the classification and regression tree (CART) algorithm [5]. This model overfits the data points when the size of the tree is too large or when each leaf-node contains an insufficient number of data points [2,5].

Supplementary Figure 8(a) and (b) show the plot of MSE and $R^2$ obtained from the decision tree algorithm respectively with an increase in the size of the trees (or maximum depth).

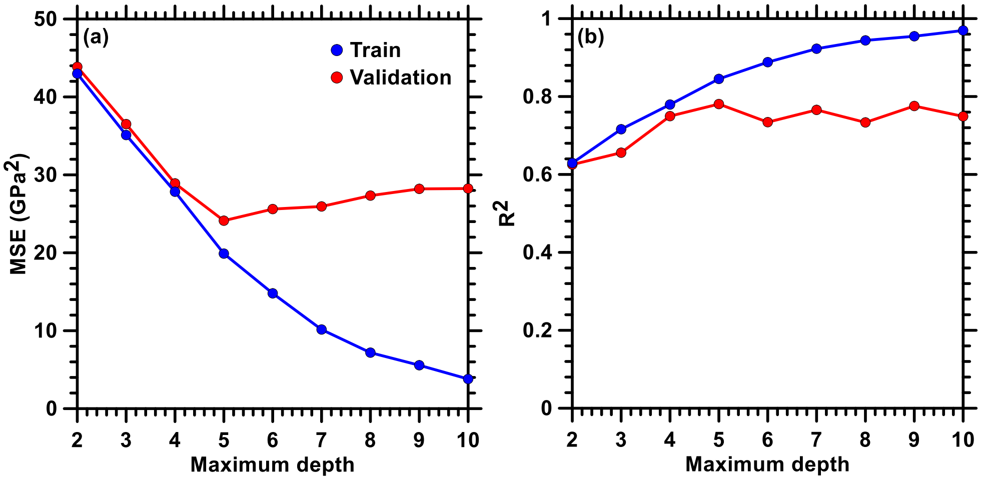


***Supplementary Figure 8**. (a) MSE and (b) $R^2$ values of the decision tree model as a function of max depth considered in each model as obtained for the train and validation set. The optimal order is chosen for such a model where minimum MSE and maximum $R^2$ of the validation set are observed.*

Here, the maximum depth characterizes the complexity of the model. It is observed that at a maximum depth of 5, a minimum MSE and maximum $R^2$of the validation data is achieved. Thus, a model complexity equals to 5 is considered as the best model for this dataset. With further increase in the model complexity, the MSE of the train set decreases however the MSE for the validation data increases. This show that the model has experienced an overfitting problem with further increase in maximum depth beyond 5, and this is also true for $R^2$ plot.

To assess the accuracy of the models, the predicted values (with max depth = 5) and the measured values from MD simulations for all the elastic constants are shown in Supplementary Figure 9 with corresponding $R^2$ values. Compared to the overall trends observed for other algorithms, here the model's prediction accuracy is comparatively lower for both the training set and test set.

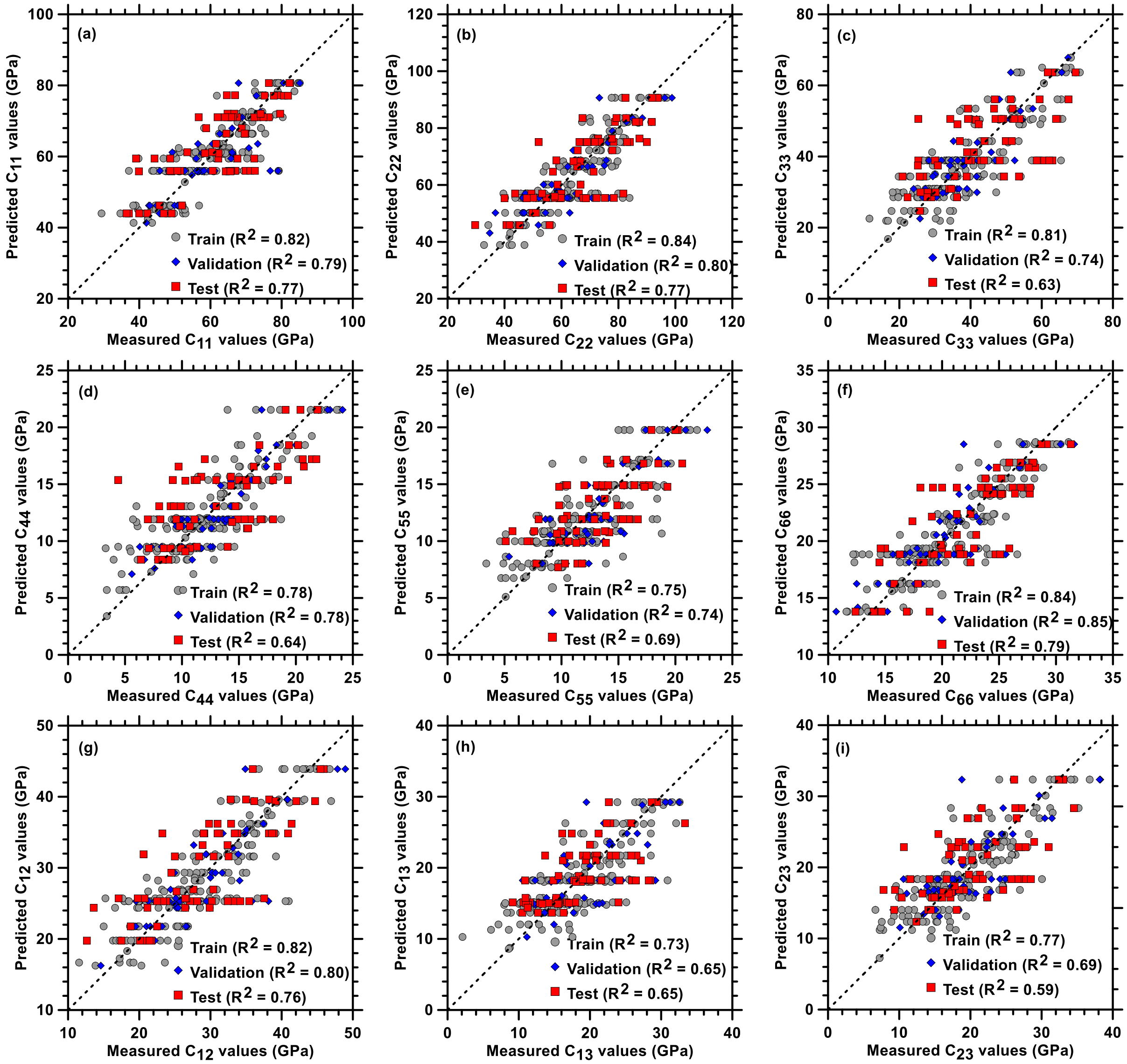


***Supplementary Figure 9.*** *Comparison of the predicted stiffness components by decision tree (with a maximum depth equal to 5) and measured values which are computed by molecular dynamics simulation.*

## Random Forest (RF)

Random forest is a decision forest or decision tree method that belongs to ensemble learning. It is an average of a large collection of decorrelated decision trees. Such an ensemble method can both increase

the prediction accuracy and reduce over-fitting problems [2]. In this model, a large number of trees are trained individually using only a subset of the input variables [2,3]. In each tree, a bootstrap sample of the training data is used instead of the entire set of train data. This procedure is known as bootstrap aggregation or bagging [2]. The predictions of each individual are then averaged to obtain the prediction of the random forest ensemble. This method is similar to boosting in many aspects but can be easily trained and manipulated.

Supplementary Figure 10(a) and (b) show the plot of MSE and $R^2$ values respectively (mean values from all nine elastic constant cases) offered by random forest algorithm with an increase in the number of trees. Here, the number of trees characterize the complexity of the model. As observed from the Supplementary Figure 10, the minimum MSE for validation set is observed when the number of trees equals to 9 and no significant change is observed for $R^2$. It is noticed that the MSE of the train set and validation set only plateaus upon increasing the number of trees and this indicates that the RF does not yield any noticeable overfitting at high model complexity.

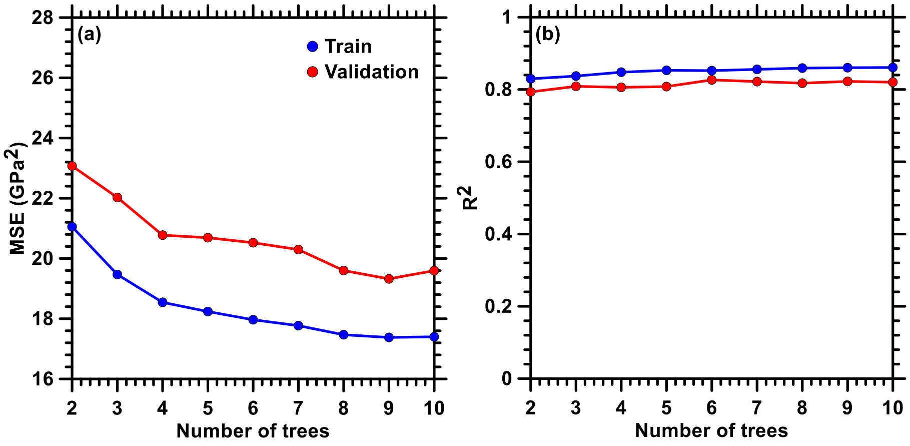


***Supplementary Figure 10****. (a) MSE and (b) $R^2$ values of the Random Forest models as a function of the number of trees considered in each model as obtained for the train and validation set. The optimal order is chosen for such a model where minimum MSE and maximum $R^2$ of the validation set are observed.*

To assess the accuracy of the models, Supplementary Figure 11 represents the prediction value obtained from the best RF model with the number of trees equal to 9 against measured values computed from MD. It is observed that the RF model performed better than the decision tree. However, it failed to accurately predict for some elastic constant such as $C_{23}$.

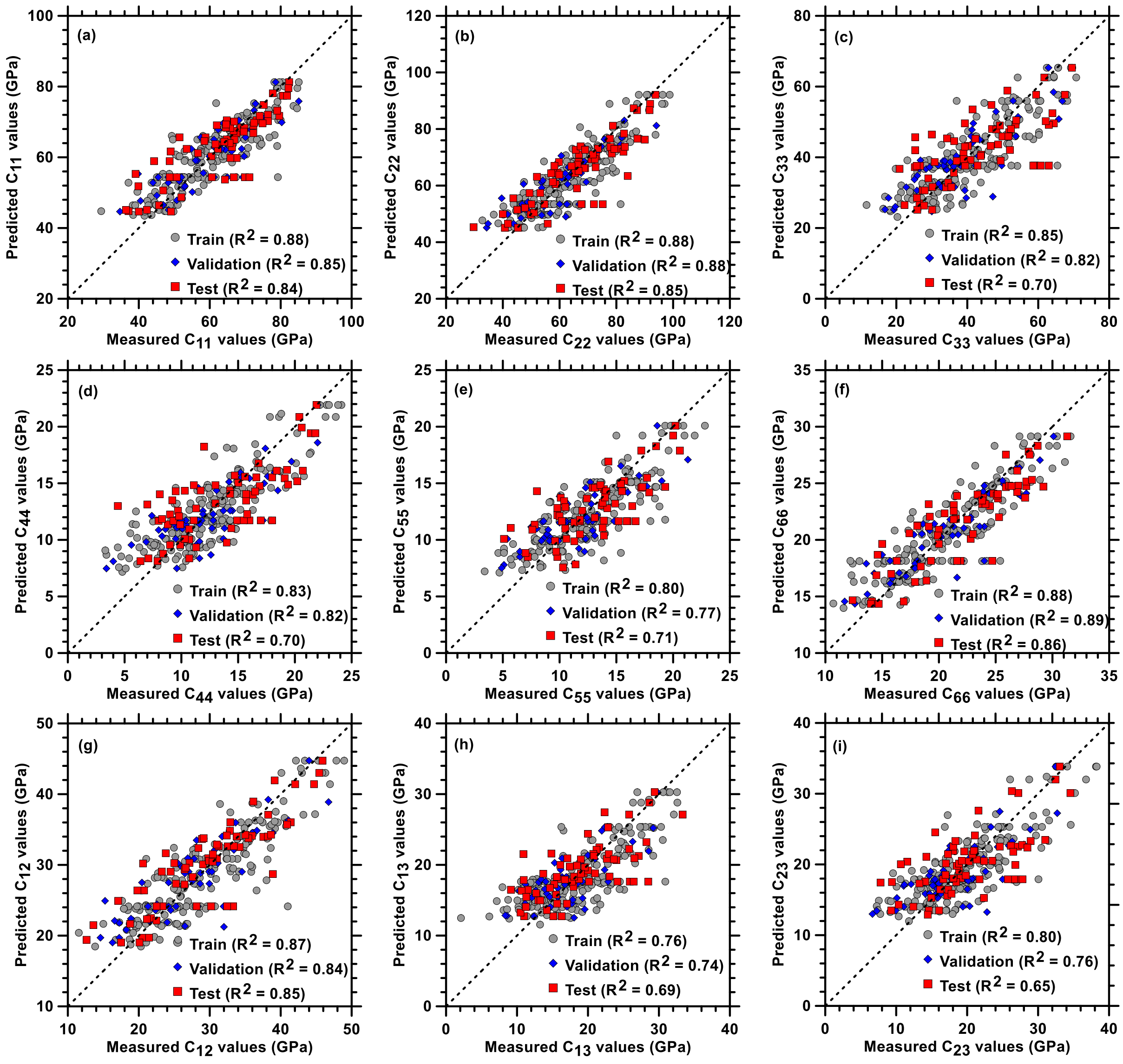


***Supplementary Figure 11.*** *Comparison of the predicted stiffness components by random forest (with number of trees equal to 9) and measured values which are computed by molecular dynamics simulation.*

**GP with Matern kernel**

Supplementary Figure 12 shows the prediction of GPR with the Matern kernel against the measured value computed by MD simulation. Here, the GPR model is trained with the train set using the marten kernel along with white noise. The model is updated till the hyperparameters converged to a global optimum. Overall, the prediction accuracies using GP with Matern kernel are found to be similar to the ones obtained using GP with rbf kernel.

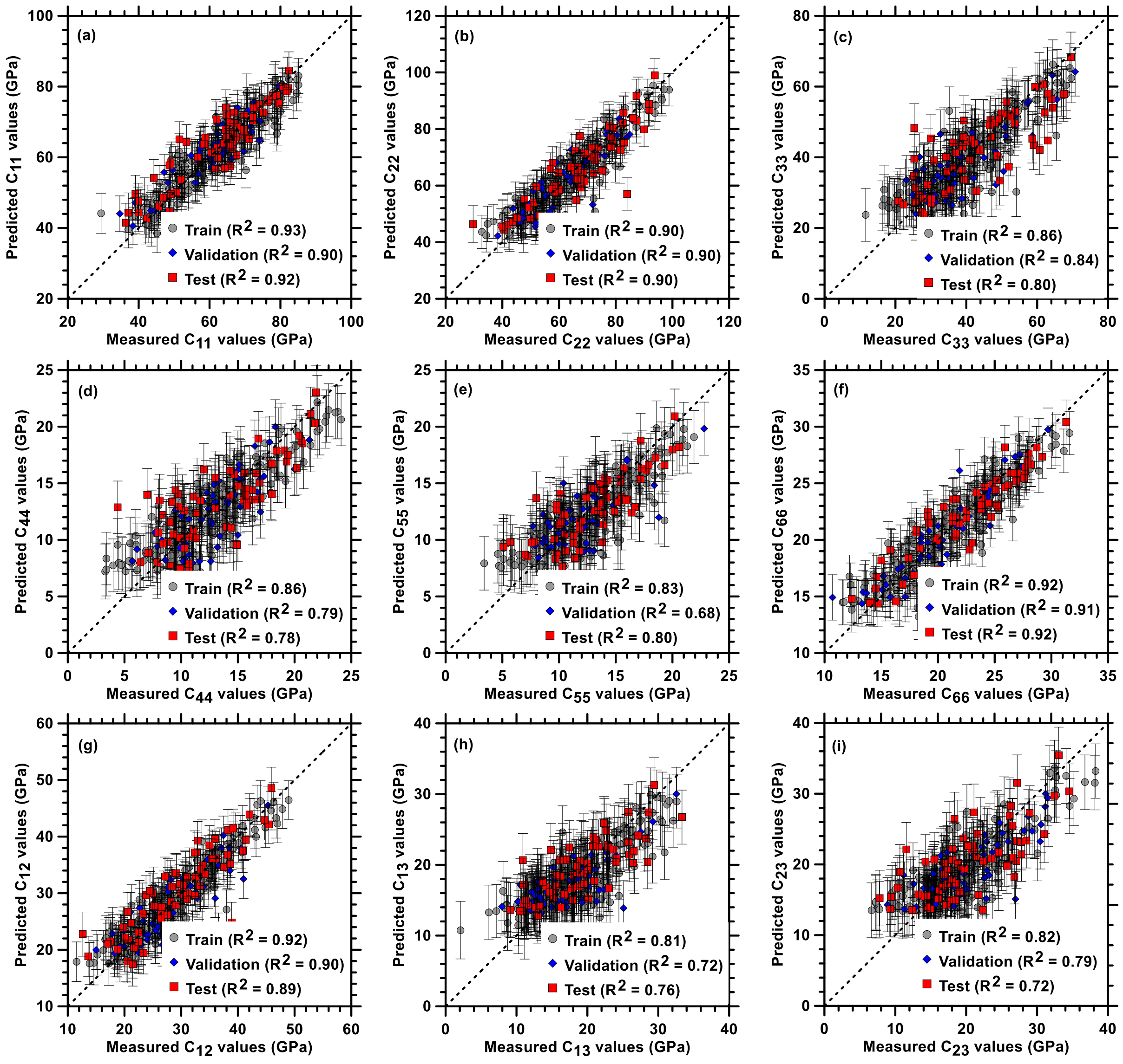


***Supplementary Figure 12.*** *Comparison of the predicted elastic constants by GPR (with Matern kernel) and measured values which are computed by molecular dynamics simulation. The error bars shown for each value represent standard deviation around the mean values.*

## Hyperparameter tuning for NN

In this work, a multilayer perceptron (MLP) approach is implemented which is a class of feedforward neural network containing an input layer, a hidden layer, and an output layer. The MLP NN model is trained using the back-propagation algorithm. Here, two hidden layers are trained with varying numbers of neurons. Supplementary Figure 13(a) and (b) plot the mean MSE and $R^2$ from all nine elastic constants obtained from the MLP-NN (with two hidden layers) with respect to increasing neurons (which characterizes the model complexity).

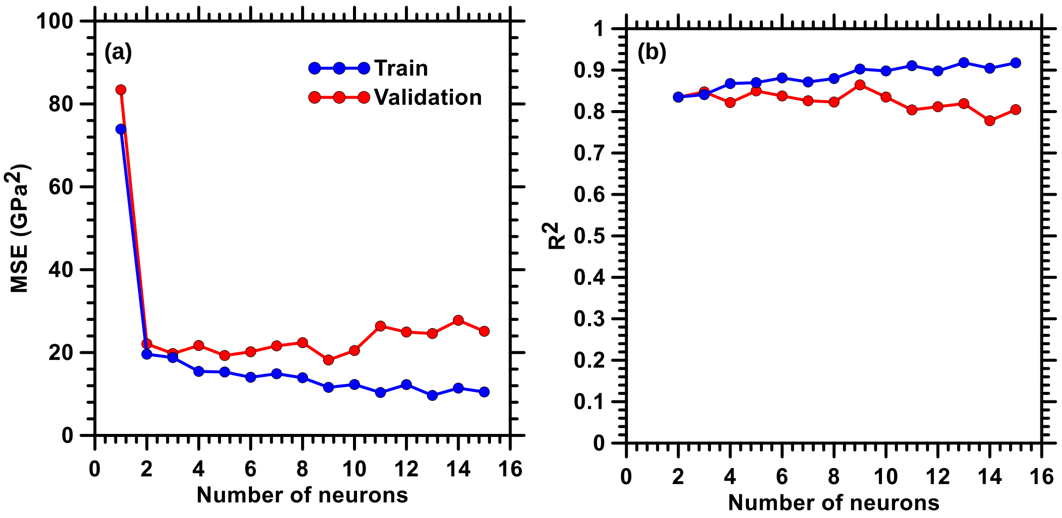


***Supplementary Figure 13****. (a) MSE and (b) $R^2$ values of the neural network as a function of the number of neurons for the train and validation set.*

Based on the trends observed in the Supplementary Figure 3(a) and 3(b), the optimum order is chosen for the model where minimum MSE and high $R^2$ for the validation set is achieved where the number of neurons is 9. The other hyperparameters that are adopted here are: (i) adam optimizer, (ii) learning rate = 5e-3, epochs = 400, and batch size = 32.

## SHAP values for elastic constants

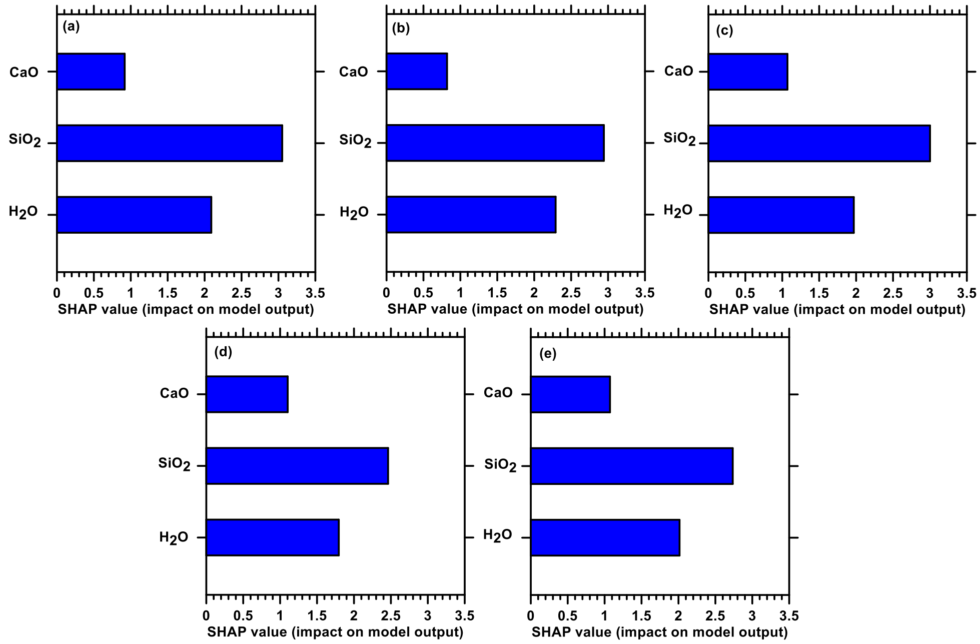


***Supplementary Figure 14.*** SHAP values for various compositions for (a) $C_{22}$, (b) $C_{55}$, (c) $C_{12}$ and (d) $C_{13}$, and (d) $C_{23}$.

## SUPPLEMENTARY REFERENCES

1. Vapnik, V. N. *Statistical learning theory*. vol. 2 (Wiley, 1998).
2. Lu, Z. Q. J. The Elements of Statistical Learning: Data Mining, Inference, and Prediction. *Journal of the Royal Statistical Society: Series A (Statistics in Society)* **173**, 693–694 (2010).
3. Kuhn, M. & Johnson, K. *Applied Predictive Modeling*. (Springer Science & Business Media, 2013).
4. Demuth, H. B., Beale, M. H., De Jess, O. & Hagan, M. T. *Neural Network Design*. (Martin Hagan, 2014).
5. Loh, W.-Y. Classification and regression trees. *WIREs Data Mining and Knowledge Discovery* **1**, 14–23 (2011).

## REAXFF POTENTIAL

Reactive MD-force field: C/Si/O/Al/Ca used in Pitman and van Duin, JACS 2012

```
 39       ! Number of general parameters
  50.0000 !Overcoordination parameter
   9.5469 !Overcoordination parameter
   1.6725 !Valency angle conjugation parameter
   1.7224 !Triple bond stabilisation parameter
   6.8702 !Triple bond stabilisation parameter
  60.4850 !C2-correction
   1.0588 !Undercoordination parameter
   4.6000 !Triple bond stabilisation parameter
  12.1176 !Undercoordination parameter
  13.3056 !Undercoordination parameter
 -55.1978 !Triple bond stabilization energy
   0.0000 !Lower Taper-radius
  10.0000 !Upper Taper-radius
   2.8793 !Not used
```

33.8667 !Valency undercoordination

6.0891 !Valency angle/lone pair parameter

1.0563 !Valency angle

2.0384 !Valency angle parameter

6.1431 !Not used

6.9290 !Double bond/angle parameter

0.3989 !Double bond/angle parameter: overcoord

3.9954 !Double bond/angle parameter: overcoord

-2.4837 !Not used

5.7796 !Torsion/BO parameter

10.0000 !Torsion overcoordination

1.9487 !Torsion overcoordination

-1.2327 !Conjugation 0 (not used)

2.1645 !Conjugation

1.5591 !vdWaals shielding

0.1000 !Cutoff for bond order (*100)

1.7602 !Valency angle conjugation parameter

0.6991 !Overcoordination parameter

50.0000 !Overcoordination parameter

1.8512 !Valency/lone pair parameter

0.5000 !Not used

20.0000 !Not used

5.0000 !Molecular energy (not used)

0.0000 !Molecular energy (not used)

0.7903 !Valency angle conjugation parameter

8 ! Nr of atoms; cov.r; valency;a.m;Rvdw;Evdw;gammaEEM;cov.r2;#

alfa;gammavdW;valency;Eunder;Eover;chiEEM;etaEEM;n.u.

cov r3;Elp;Heat inc.;n.u.;n.u.;n.u.;n.u.

ov/un;val1;n.u.;val3,vval4

| | | | | | | | | |
|---|---|---|---|---|---|---|---|---|
| C | 1.3817 | 4.0000 | 12.0000 | 1.8903 | 0.1838 | 0.9000 | 1.1341 | 4.0000 |
| | 9.7559 | 2.1346 | 4.0000 | 34.9350 | 79.5548 | 5.9666 | 7.0000 | 0.0000 |
| | 1.2114 | 0.0000 | 202.6057 | 8.9539 | 34.9289 | 13.5366 | 0.8563 | 0.0000 |
| | -2.8983 | 2.5000 | 1.0564 | 4.0000 | 2.9663 | 1.2000 | 0.2000 | 13.0000 |
| H | 0.8930 | 1.0000 | 1.0080 | 1.3550 | 0.0930 | 0.8203 | -0.1000 | 1.0000 |
| | 8.2230 | 33.2894 | 1.0000 | 0.0000 | 121.1250 | 3.7248 | 9.6093 | 1.0000 |
| | -0.1000 | 0.0000 | 61.6606 | 3.0408 | 2.4197 | 0.0003 | 1.0698 | 0.0000 |
| | -19.4571 | 4.2733 | 1.0338 | 1.0000 | 2.8793 | 1.0000 | 0.2000 | 12.0000 |
| O | 1.2450 | 2.0000 | 15.9990 | 2.3890 | 0.1000 | 1.0898 | 1.0548 | 6.0000 |
| | 9.7300 | 13.8449 | 4.0000 | 37.5000 | 116.0768 | 8.5000 | 8.3122 | 2.0000 |
| | 0.9049 | 0.4056 | 59.0626 | 3.5027 | 0.7640 | 0.0021 | 0.9745 | 0.0000 |
| | -3.5500 | 2.9000 | 1.0493 | 4.0000 | 2.9225 | 1.3000 | 0.2000 | 13.0000 |
| Fe | 1.9506 | 3.0000 | 55.8450 | 2.0308 | 0.1274 | 0.7264 | -1.0000 | 3.0000 |
| | 11.0534 | 2.2637 | 3.0000 | 0.0000 | 18.3725 | 1.2457 | 7.3021 | 0.0000 |
| | -1.2000 | 0.0000 | 66.4838 | 30.0000 | 1.0000 | 0.0000 | 0.8563 | 0.0000 |
| | -16.2040 | 2.7917 | 1.0338 | 6.0000 | 2.5791 | 1.3000 | 0.2000 | 13.0000 |
| Cl | 1.7140 | 1.0000 | 35.4500 | 1.9139 | 0.2000 | 0.3837 | -1.0000 | 7.0000 |
| | 11.5345 | 10.1330 | 1.0000 | 0.0000 | 0.0000 | 9.9614 | 6.5316 | 0.0000 |
| | -1.0000 | 3.5750 | 143.1770 | 6.2293 | 5.2294 | 0.1542 | 0.8563 | 0.0000 |
| | -10.2080 | 2.9867 | 1.0338 | 6.2998 | 2.5791 | 1.3000 | 0.2000 | 13.0000 |
| Si | 2.1932 | 4.0000 | 28.0600 | 1.8951 | 0.1737 | 0.8112 | 1.2962 | 4.0000 |
| | 11.3429 | 5.2054 | 4.0000 | 21.7115 | 139.9309 | 4.0081 | 5.7104 | 0.0000 |
| | -1.0000 | 0.0000 | 128.2031 | 9.0751 | 23.8188 | 0.8381 | 0.8563 | 0.0000 |
| | -4.1684 | 2.0754 | 1.0338 | 4.0000 | 2.5791 | 1.4000 | 0.2000 | 13.0000 |
| Al | 2.1967 | 3.0000 | 26.9820 | 2.3738 | 0.2328 | 0.4558 | -1.6836 | 3.0000 |
| | 9.4002 | 3.9009 | 3.0000 | 0.0076 | 16.5151 | 1.6032 | 6.7003 | 0.0000 |
| | -1.0000 | 0.0000 | 78.4675 | 20.0000 | 0.2500 | 0.0000 | 0.8563 | 0.0000 |

-23.1826 1.5000 1.0338 8.0000 2.5791 1.4000 0.2000 13.0000

Ca 1.9927 2.0000 40.0870 2.7005 0.1848 0.7939 1.0000 2.0000

10.6123 27.5993 3.0000 38.0000 0.0000 -1.9372 6.5275 0.0000

-1.3000 0.0000 220.0000 49.9248 0.3370 0.0000 0.0000 0.0000

-2.0000 4.0000 1.0564 6.2998 2.9663 1.4000 0.0100 13.0000

28 ! Nr of bonds; Edis1;LPpen;n.u.;pbe1;pbo5;13corr;pbo6

pbe2;pbo3;pbo4;Etrip;pbo1;pbo2;ovcorr

1 1 158.2004 99.1897 78.0000 -0.7738 -0.4550 1.0000 37.6117 0.4147

0.4590 -0.1000 9.1628 1.0000 -0.0777 6.7268 1.0000 0.0000

1 2 169.4760 0.0000 0.0000 -0.6083 0.0000 1.0000 6.0000 0.7652

5.2290 1.0000 0.0000 1.0000 -0.0500 6.9136 0.0000 0.0000

2 2 153.3934 0.0000 0.0000 -0.4600 0.0000 1.0000 6.0000 0.7300

6.2500 1.0000 0.0000 1.0000 -0.0790 6.0552 0.0000 0.0000

1 3 164.4303 82.6772 60.8077 -0.3739 -0.2351 1.0000 10.5036 1.0000

0.4475 -0.2288 7.0250 1.0000 -0.1363 4.8734 0.0000 0.0000

3 3 142.2858 145.0000 50.8293 0.2506 -0.1000 1.0000 29.7503 0.6051

0.3451 -0.1055 9.0000 1.0000 -0.1225 5.5000 1.0000 0.0000

2 3 160.0000 0.0000 0.0000 -0.5725 0.0000 1.0000 6.0000 0.5626

1.1150 1.0000 0.0000 0.0000 -0.0920 4.2790 0.0000 0.0000

1 4 133.0514 0.0000 0.0000 1.0000 -0.3000 1.0000 36.0000 0.0673

0.2350 -0.3500 15.0000 1.0000 -0.1143 4.5217 1.0000 0.0000

2 4 105.0054 0.0000 0.0000 -0.0717 0.0000 0.0000 6.0000 0.0505

0.1000 1.0000 0.0000 1.0000 -0.1216 4.5062 0.0000 0.0000

3 4 65.7713 0.0000 0.0000 0.1366 -0.3000 1.0000 36.0000 0.0494

0.9495 -0.3500 15.0000 1.0000 -0.0555 7.9897 1.0000 0.0000

4 4 38.7471 0.0000 0.0000 0.3595 -0.2000 0.0000 16.0000 0.2749

1.0000 -0.2000 15.0000 1.0000 -0.0771 6.4477 0.0000 0.0000

2 5 109.1686 0.0000 0.0000 -0.1657 -0.2000 0.0000 16.0000 1.2500

2.8463 -0.2000 15.0000 1.0000 -0.1111 5.2687 0.0000 0.0000

3 5 0.0000 0.0000 0.0000 0.5000 -0.2000 0.0000 16.0000 0.5000

1.0001 -0.2000 15.0000 1.0000 -0.1000 10.0000 0.0000 0.0000

4 5 0.0000 0.0000 0.0000 0.2500 -0.2000 0.0000 16.0000 0.5000

0.5000 -0.2000 15.0000 1.0000 -0.2000 10.0000 0.0000 0.0000

5 5 0.2500 0.0000 0.0000 0.1803 -0.2000 0.0000 16.0000 0.3356

0.9228 -0.2000 15.0000 1.0000 -0.1178 5.6715 0.0000 0.0000

1 6 0.0000 0.0000 0.0000 -0.6528 -0.3000 0.0000 36.0000 0.5000

10.0663 -0.3500 25.0000 1.0000 -0.1000 10.0000 0.0000 0.0000

2 6 250.0000 0.0000 0.0000 -0.7128 0.0000 1.0000 6.0000 0.1186

18.5790 1.0000 0.0000 1.0000 -0.0731 7.4983 0.0000 0.0000

3 6 261.9074 5.9533 0.0000 -0.6223 -0.3000 1.0000 36.0000 0.7275

10.1541 -0.2366 29.7817 1.0000 -0.1083 8.5924 6.0658 0.0000

6 6 70.9120 54.0531 30.0000 0.4931 -0.3000 1.0000 16.0000 0.0392

0.2476 -0.8055 7.1248 1.0000 -0.1009 8.7229 0.0000 0.0000

1 7 0.0000 0.0000 0.0000 -0.6528 -0.3000 0.0000 36.0000 0.5000

10.0663 -0.3500 25.0000 1.0000 -0.1000 10.0000 0.0000 0.0000

2 7 92.8579 0.0000 0.0000 -0.6528 -0.3000 0.0000 36.0000 0.1551

10.0663 -0.3500 25.0000 1.0000 -0.0842 7.1758 0.0000 0.0000

3 7 228.4876 0.0000 0.0000 -0.8524 -0.3000 0.0000 36.0000 0.1252

0.4016 -0.3500 25.0000 1.0000 -0.1750 5.2102 0.0000 0.0000

6 7 0.0000 0.0000 0.0000 1.0000 0.3000 0.0000 26.0000 1.0000

0.5000 0.0000 12.0000 1.0000 -0.2000 10.0000 0.0000 0.0000

7 7 34.0777 0.0000 0.0000 0.4832 -0.3000 0.0000 16.0000 0.5154

6.4631 -0.4197 14.3085 1.0000 -0.1463 6.1608 0.0000 0.0000

2 8 0.0000 0.0000 0.0000 -0.0203 -0.1418 1.0000 13.1260 0.0230

8.2136 -0.1310 0.0000 1.0000 -0.2692 6.4254 0.0000 24.4461

3 8 50.8757 0.0000 43.3991 1.0000 -0.3000 1.0000 36.0000 0.0025

```
      0.7609  -0.2500  12.0000   1.0000  -0.0515   8.9041   1.0000  24.4461
  6  8   0.0000   0.0000   0.0000   0.5000  -0.3000   1.0000  16.0000   0.5000
      0.5000  -0.2500  15.0000   1.0000  -0.1000   9.0000   0.0000   0.0000
  7  8   0.0000   0.0000   0.0000   0.5000  -0.3000   1.0000  16.0000   0.5000
      0.5000  -0.2500  15.0000   1.0000  -0.1000   9.0000   0.0000   0.0000
  8  8  36.9494   0.0000   0.0000  -0.0412  -0.2000   0.0000  16.0000   0.3233
      0.3708  -0.2000  10.0000   1.0000  -0.0822   4.2104   0.0000   0.0000
21    ! Nr of off-diagonal terms; Ediss;Ro;gamma;rsigma;rpi;rpi2
  1  2   0.1239   1.4004   9.8467   1.1210  -1.0000  -1.0000
  2  3   0.0283   1.2885  10.9190   0.9215  -1.0000  -1.0000
  1  3   0.1345   1.8422   9.7725   1.2835   1.1576   1.0637
  1  4   0.1358   1.8293  10.0425   1.6096  -1.0000  -1.0000
  2  4   0.0640   1.6974  11.5167   1.3517  -1.0000  -1.0000
  3  4   0.0846   1.4284  10.0808   1.8339  -1.0000  -1.0000
  2  5   0.0568   1.6740   9.6297   1.2200  -1.0000  -1.0000
  3  5   0.1927   2.2551  11.2308  -1.0000  -1.0000  -1.0000
  4  5   0.1500   2.1500  11.0000  -1.0000  -1.0000  -1.0000
  1  6   0.2000   1.9000  12.0000  -1.0000  -1.0000  -1.0000
  2  6   0.2000   1.5207  12.9535   1.2125  -1.0000  -1.0000
  3  6   0.2000   1.9048  10.8374   1.7163   1.2444  -1.0000
  1  7   0.2000   1.9000  12.0000  -1.0000  -1.0000  -1.0000
  2  7   0.0564   1.4937  12.0744   1.7276  -1.0000  -1.0000
  3  7   0.1651   1.8998  11.2212   1.5416  -1.0000  -1.0000
  6  7   0.0216   1.5025  11.8792  -1.0000  -1.0000  -1.0000
  1  8   0.1000   1.9000  11.5000  -1.0000  -1.0000  -1.0000
  2  8   0.0100   1.6000  13.2979  -1.0000  -1.0000  -1.0000
  3  8   0.0955   1.7587  11.9417   1.9052  -1.0000  -1.0000
  6  8   0.1000   1.9000  11.0000  -1.0000  -1.0000  -1.0000
```

7 8 0.1000 1.9000 11.0000 -1.0000 -1.0000 -1.0000

68 ! Nr of angles;at1;at2;at3;Thetao,o;ka;kb;pv1;pv2

1 1 1 59.0573 30.7029 0.7606 0.0000 0.7180 6.2933 1.1244

1 1 2 65.7758 14.5234 6.2481 0.0000 0.5665 0.0000 1.6255

2 1 2 70.2607 25.2202 3.7312 0.0000 0.0050 0.0000 2.7500

1 2 2 0.0000 0.0000 6.0000 0.0000 0.0000 0.0000 1.0400

1 2 1 0.0000 3.4110 7.7350 0.0000 0.0000 0.0000 1.0400

2 2 2 0.0000 27.9213 5.8635 0.0000 0.0000 0.0000 1.0400

1 1 3 53.9517 7.8968 2.6122 0.0000 3.0000 58.6562 1.0338

3 1 3 76.9627 44.2852 2.4177 -25.3063 1.6334 -50.0000 2.7392

2 1 3 65.0000 16.3141 5.2730 0.0000 0.4448 0.0000 1.4077

1 3 1 72.6199 42.5510 0.7205 0.0000 2.9294 0.0000 1.3096

1 3 3 81.9029 32.2258 1.7397 0.0000 0.9888 68.1072 1.7777

3 3 3 80.7324 30.4554 0.9953 0.0000 3.0000 50.0000 1.0783

1 3 2 70.1101 13.1217 4.4734 0.0000 0.8433 0.0000 3.0000

2 3 3 75.6935 50.0000 2.0000 0.0000 1.0000 0.0000 1.1680

2 3 2 85.8000 9.8453 2.2720 0.0000 2.8635 0.0000 1.5800

1 2 3 0.0000 25.0000 3.0000 0.0000 1.0000 0.0000 1.0400

3 2 3 0.0000 15.0000 2.8900 0.0000 0.0000 0.0000 2.8774

2 2 3 0.0000 8.5744 3.0000 0.0000 0.0000 0.0000 1.0421

1 4 1 29.1655 3.3035 0.2000 0.0000 1.1221 0.0000 1.0562

1 1 4 59.8697 2.8115 1.9262 0.0000 0.7602 0.0000 1.4056

1 4 4 25.4591 15.9430 0.9664 0.0000 2.2242 0.0000 1.1088

4 1 4 88.6279 26.0015 1.0328 0.0000 0.2361 0.0000 2.0576

2 1 4 47.3695 16.9204 4.1052 0.0000 0.1000 0.0000 1.0050

2 4 2 34.1965 6.6782 6.5943 0.0000 1.3895 0.0000 1.5365

2 2 4 0.1000 30.0000 3.4094 0.0000 2.4379 0.0000 1.5166

4 2 4 0.0000 8.2994 5.7832 0.0000 2.9873 0.0000 1.7716

| | | | | | | | | | |
|---|---|---|---|---|---|---|---|---|---|
| 2 | 4 | 4 | 21.2590 | 6.5954 | 0.9951 | 0.0000 | 2.8006 | 0.0000 | 1.0000 |
| 2 | 4 | 4 | 180.0000 | -6.9970 | 24.3956 | 0.0000 | 0.7878 | 0.0000 | 1.3672 |
| 1 | 3 | 4 | 90.0000 | 12.8684 | 1.4601 | 0.0000 | 0.8757 | 0.0000 | 1.0000 |
| 3 | 1 | 4 | 18.8567 | 24.3753 | 3.9647 | 0.0000 | 0.1000 | 0.0000 | 1.5314 |
| 3 | 4 | 3 | 79.7335 | 0.0100 | 0.1392 | 0.0000 | 0.4968 | 0.0000 | 2.1948 |
| 4 | 3 | 4 | 57.6787 | 4.8566 | 2.5768 | 0.0000 | 0.7552 | 0.0000 | 1.0000 |
| 2 | 3 | 4 | 59.4556 | 10.2025 | 0.7481 | 0.0000 | 1.4521 | 0.0000 | 1.0000 |
| 3 | 3 | 4 | 73.6721 | 32.6330 | 1.7223 | 0.0000 | 1.0221 | 0.0000 | 1.4351 |
| 3 | 4 | 4 | 65.7545 | 5.6268 | 4.0645 | 0.0000 | 1.7794 | 0.0000 | 2.6730 |
| 3 | 2 | 4 | 0.0000 | 4.6026 | 2.5343 | 0.0000 | 0.7284 | 0.0000 | 1.1051 |
| 2 | 4 | 3 | 34.0653 | 20.1868 | 4.7461 | 0.0000 | 0.1000 | 0.0000 | 1.6752 |
| 3 | 2 | 5 | 0.0000 | 0.0100 | 0.5211 | 0.0000 | 0.0000 | 0.0000 | 1.3859 |
| 6 | 6 | 6 | 78.5339 | 36.4328 | 1.0067 | 0.0000 | 0.1694 | 0.0000 | 1.6608 |
| 2 | 6 | 6 | 77.2616 | 5.0190 | 7.8944 | 0.0000 | 4.0000 | 0.0000 | 1.0400 |
| 2 | 6 | 2 | 75.7983 | 14.4132 | 2.8640 | 0.0000 | 4.0000 | 0.0000 | 1.0400 |
| 3 | 6 | 6 | 90.6812 | 31.1846 | 4.4543 | 0.0000 | 0.5073 | 0.0000 | 2.1809 |
| 2 | 6 | 3 | 73.6998 | 40.0000 | 1.8782 | 0.0000 | 4.0000 | 0.0000 | 1.1290 |
| 3 | 6 | 3 | 80.1361 | 36.2368 | 0.9504 | 0.0000 | 0.2624 | 0.0000 | 2.0787 |
| 6 | 3 | 6 | 80.4450 | 6.0739 | 1.7731 | 0.0000 | 3.2548 | 0.0000 | 1.0422 |
| 2 | 3 | 6 | 86.7611 | 7.1742 | 1.4013 | 0.0000 | 1.4999 | 0.0000 | 1.0400 |
| 3 | 3 | 6 | 103.4529 | 26.9589 | 1.3470 | 0.0000 | 1.7728 | 0.0000 | 1.3091 |
| 2 | 2 | 6 | 0.0000 | 47.1300 | 6.0000 | 0.0000 | 1.6371 | 0.0000 | 1.0400 |
| 6 | 2 | 6 | 0.0000 | 27.4206 | 6.0000 | 0.0000 | 1.6371 | 0.0000 | 1.0400 |
| 3 | 2 | 6 | 0.0000 | 5.0000 | 1.0000 | 0.0000 | 1.0000 | 0.0000 | 1.2500 |
| 3 | 2 | 7 | 0.0000 | 4.2750 | 1.0250 | 0.0000 | 1.3750 | 0.0000 | 1.4750 |
| 2 | 2 | 7 | 0.0000 | 3.0000 | 1.0000 | 0.0000 | 1.0000 | 0.0000 | 1.2500 |
| 7 | 2 | 7 | 0.0000 | 20.2391 | 0.1328 | 0.0000 | 2.9860 | 0.0000 | 1.0870 |
| 2 | 3 | 7 | 88.1144 | 13.2143 | 1.5068 | 0.0000 | 3.0000 | 0.0000 | 1.0100 |

3 3 7 34.4326 25.9544 5.1239 0.0000 2.7500 0.0000 1.7141

7 3 7 21.6945 20.0000 4.0000 0.0000 0.6619 0.0000 1.9714

2 7 2 67.4229 4.5148 5.9702 0.0000 3.0000 0.0000 2.6879

2 7 3 41.8108 17.3800 2.6618 0.0000 0.7372 0.0000 1.0100

3 7 3 49.1145 11.8902 2.1383 0.0000 3.0000 0.0000 1.4790

2 7 7 180.0000 -26.7860 7.3549 0.0000 1.0000 0.0000 1.0252

2 7 7 78.2279 37.6504 0.4809 0.0000 1.0000 0.0000 2.9475

6 3 7 16.5023 0.0100 2.7027 0.0000 1.0000 0.0000 1.0000

3 6 7 88.2703 0.3954 0.2500 0.0000 0.5000 0.0000 2.1060

3 7 6 83.8306 0.3712 0.2500 0.0000 0.5000 0.0000 2.1153

3 8 3 1.0000 4.9611 2.4541 0.0000 0.5754 0.0000 1.0000

8 3 8 9.5066 4.2640 3.1438 0.0000 1.9819 0.0000 1.6463

2 3 8 51.3829 2.5000 0.2500 0.0000 0.0500 0.0000 1.0000

3 3 8 70.0000 25.0000 1.0000 0.0000 1.0000 0.0000 1.2500

32 ! Nr of torsions;at1;at2;at3;at4;;V1;V2;V3;V2(BO);vconj;n.u;n

1 1 1 1 -0.2500 34.7453 0.0288 -6.3507 -1.6000 0.0000 0.0000

1 1 1 2 -0.2500 29.2131 0.2945 -4.9581 -2.1802 0.0000 0.0000

2 1 1 2 -0.2500 31.2081 0.4539 -4.8923 -2.2677 0.0000 0.0000

1 1 1 3 1.2799 20.7787 -0.5249 -2.5000 -1.0000 0.0000 0.0000

2 1 1 3 1.9159 19.8113 0.7914 -4.6995 -1.0000 0.0000 0.0000

3 1 1 3 -1.4477 16.6853 0.6461 -4.9622 -1.0000 0.0000 0.0000

1 1 3 1 0.4816 19.6316 -0.0057 -2.5000 -1.0000 0.0000 0.0000

1 1 3 2 1.2044 80.0000 -0.3139 -6.1481 -1.0000 0.0000 0.0000

2 1 3 1 -2.5000 31.0191 0.6165 -2.7733 -2.9807 0.0000 0.0000

2 1 3 2 -2.4875 70.8145 0.7582 -4.2274 -3.0000 0.0000 0.0000

1 1 3 3 -0.3566 10.0000 0.0816 -2.6110 -1.9631 0.0000 0.0000

2 1 3 3 -1.4383 80.0000 1.0000 -3.6877 -2.8000 0.0000 0.0000

3 1 3 1 -1.1390 78.0747 -0.0964 -4.5172 -3.0000 0.0000 0.0000

```
3  1  3  2  -2.5000  70.3345  -1.0000  -5.5315  -3.0000   0.0000   0.0000
3  1  3  3  -2.0234  80.0000   0.1684  -3.1568  -2.6174   0.0000   0.0000
1  3  3  1   1.1637 -17.3637   0.5459  -3.6005  -2.6938   0.0000   0.0000
1  3  3  2  -2.1289  12.8382   1.0000  -5.6657  -2.9759   0.0000   0.0000
2  3  3  2   2.5000 -22.9397   0.6991  -3.3961  -1.0000   0.0000   0.0000
1  3  3  3   2.5000 -25.0000   1.0000  -2.5000  -1.0000   0.0000   0.0000
2  3  3  3  -2.5000  -2.5103  -1.0000  -2.5000  -1.0000   0.0000   0.0000
3  3  3  3  -2.5000 -25.0000   1.0000  -2.5000  -1.0000   0.0000   0.0000
0  1  2  0   0.0000   0.0000   0.0000   0.0000   0.0000   0.0000   0.0000
0  2  2  0   0.0000   0.0000   0.0000   0.0000   0.0000   0.0000   0.0000
0  2  3  0   0.0000   0.1000   0.0200  -2.5415   0.0000   0.0000   0.0000
0  1  1  0   0.0000  50.0000   0.3000  -4.0000  -2.0000   0.0000   0.0000
0  3  3  0   0.5511  25.4150   1.1330  -5.1903  -1.0000   0.0000   0.0000
1  1  3  3  -0.0002  20.1851   0.1601  -9.0000  -2.0000   0.0000   0.0000
1  3  3  1   0.0002  80.0000  -1.5000  -4.4848  -2.0000   0.0000   0.0000
3  1  3  3  -0.1583  20.0000   1.5000  -9.0000  -2.0000   0.0000   0.0000
2  6  6  2   0.0000   0.0000   0.0640  -2.4426   0.0000   0.0000   0.0000
2  6  6  6   0.0000   0.0000   0.1587  -2.4426   0.0000   0.0000   0.0000
0  2  6  0   0.0000   0.0000   0.1200  -2.4847   0.0000   0.0000   0.0000
1    ! Nr of hydrogen bonds;at1;at2;at3;Rhb;Dehb;vhb1
3  2  3   2.1200  -3.5800   1.4500  19.5000
```
